# Magnetic resonance imaging of sellar and juxtasellar abnormalities in the paediatric population: an imaging review

**DOI:** 10.1007/s13244-015-0401-5

**Published:** 2015-03-21

**Authors:** Rachel Shields, Rajiv Mangla, Jeevak Almast, Steven Meyers

**Affiliations:** Department of Imaging Sciences, University of Rochester Medical Center, 601 Elmwood Ave., Rochester, NY 14642-0001 USA

**Keywords:** Magnetic resonance imaging, Paediatrics, Pituitary gland, Pituitary diseases, Sella turcica

## Abstract

The sellar and juxtasellar regions in the paediatric population are complex both anatomically and pathologically, with magnetic resonance imaging (MRI) being the “gold standard” imaging modality due to the high contrast of detail. Assessment requires a detailed understanding of the anatomy, embryology, pathophysiology and normal signal characteristics of the pituitary gland and surrounding structures in order to appropriately characterise abnormalities. This article aims to provide an overview of the imaging characteristics of developmental/congenital and acquired disease processes which affect the sellar and juxtasellar region in the paediatric population.

*Main Messages*

• *The sellar region is anatomically complex and covers a wide pathology spectrum.*

• *MRI is the key imaging modality to assess sellar and juxtasellar pathology.*

• *Numerous developmental anomalies may not be discovered until adulthood.*

• *Knowledge of pathology alerts and guides the clinician towards appropriate management.*

## Pituitary function and anatomy

**Fig. 1:**
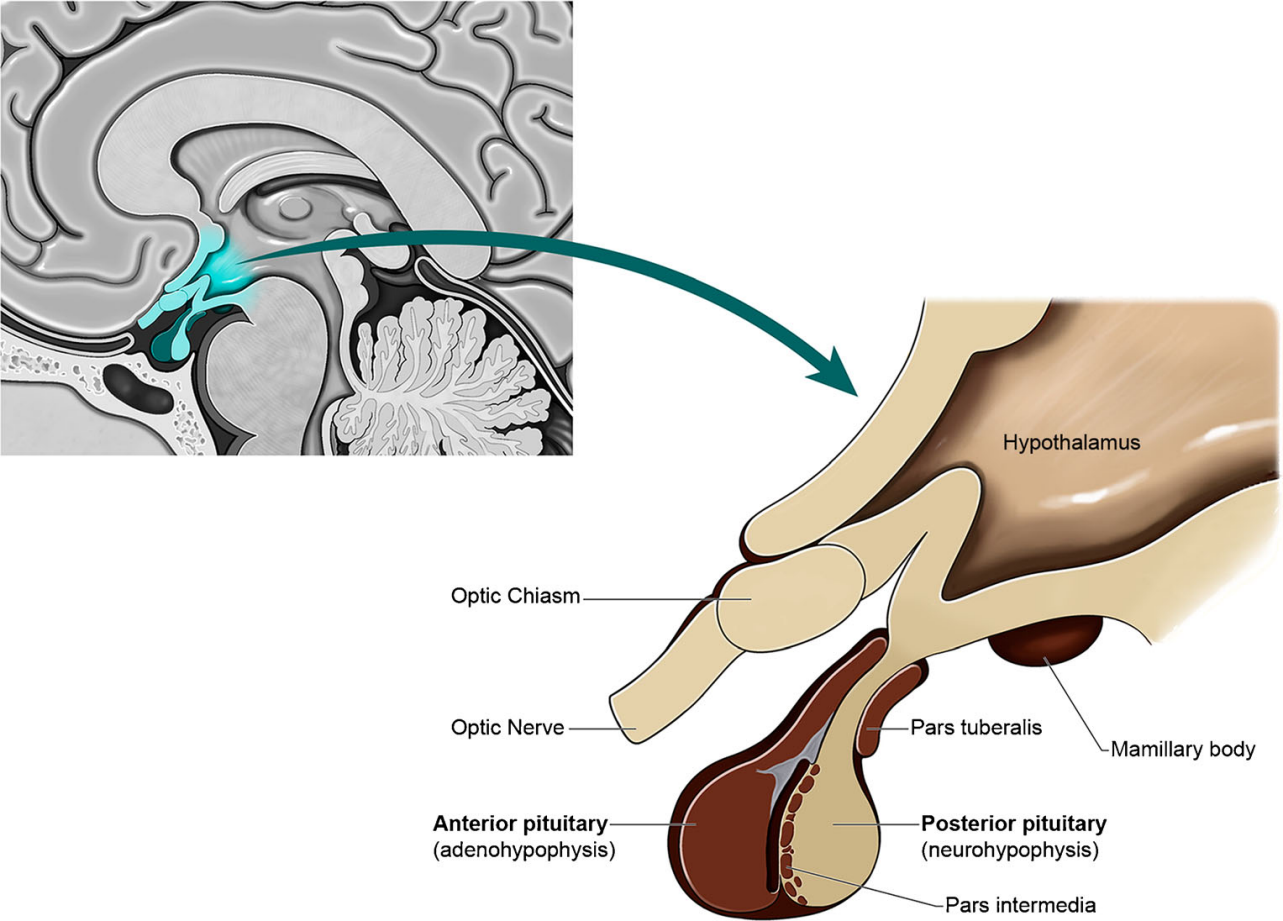
Normal pituitary anatomy

The pituitary gland is comprised of two dominant lobes, the neurohypophysis and the adenohypophysis. It is situated within the sella turcica, a depression in the sphenoid bone which is covered by the diaphragm sella with a defect for the passage of the infundibulum. The pituitary stalk extends inferiorly as a continuation of the hypothalamic infundibulum (Fig. 1).

The neurohypophysis receives its hormonal supply through direct contiguity with the hypothalamus, with axons extending from the hypothalamic supraoptic and paraventricular nuclei into the neurohypophysis. The hormonal supply includes oxytocin and vasopressin-containing vesicles. The adenohypophysis does not have axonal contiguity between the hypothalamus and the anterior gland and relies on the hypothalamic-hypophyseal portal system. Parvocellular messengers are transported from the hypothalamic median eminence into the capillaries surrounding the anterior gland. Hormones from the adenohypophysis include: follicle stimulating hormone, luteinising hormone, corticotropin (ACTH), thyroid-stimulating hormone, prolactin and growth hormone.

## Embryology

**Fig. 2:**
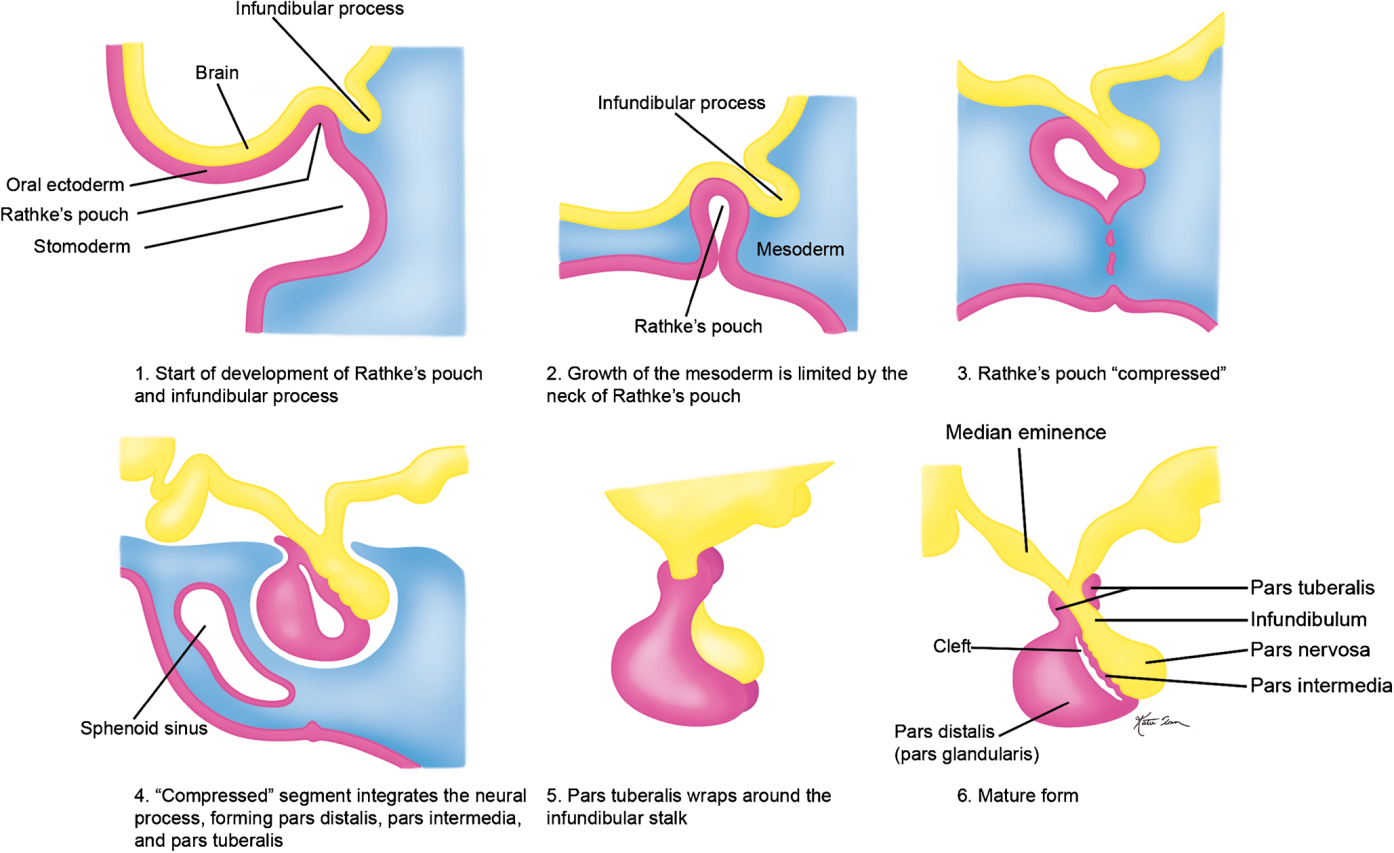
Normal development of the pituitary gland

The pituitary gland develops through a complex mechanism involving the developing diencephalon and the primitive oral stomodeum [1]. The adenohypophysis derives from Rathke’s pouch, which originates rostral to the oropharyngeal membrane. This begins at approximately 24 days of gestation, in which Rathke’s pouch forms as an ectodermal outpouching of stomodeum and the infundibulum which forms in the floor of the diencephalon. The infundibulum grows ventrally, while simultaneously Rathke’s pouch grows dorsally. Rathke’s pouch eventually loses its connection with the stomodeum and forms a sac which adheres to the infundibular process. This sac continues to differentiate into the adenophyophysis and the distal aspect of the infundibulum differentiates to form the neurohypophysis (Fig. 2).

## Imaging characteristics in the paediatric population

**Fig. 3:**
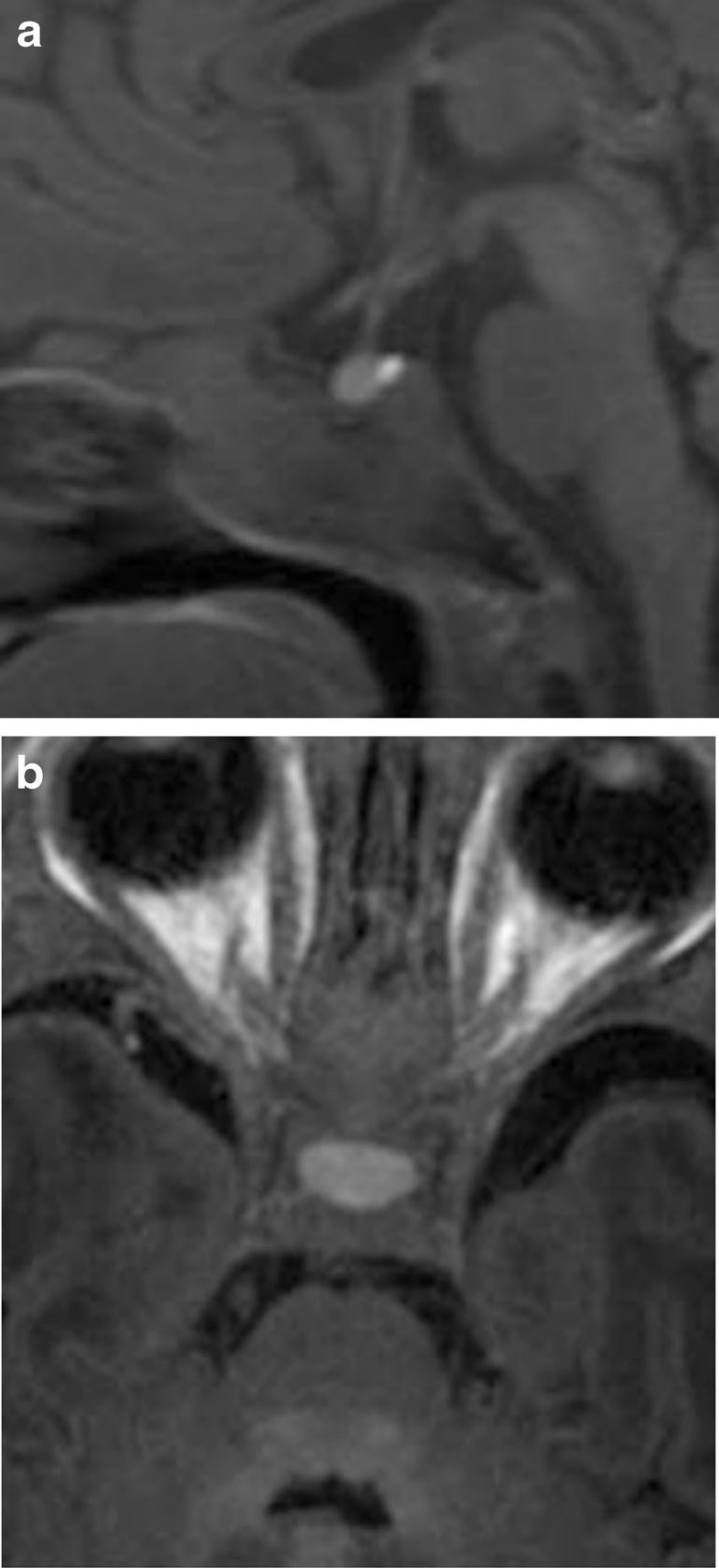
Normal physiological T1 sagittal (**a**) and axial (**b**) hyperintensity of the anterior pituitary in a 1-month-old baby

The pituitary gland exhibits a wide range of appearances during the first 2 years of life, with considerable variability in size, shape, and signal. At birth, both the anterior and posterior pituitary gland have high signal intensity on T1-weighted images (Fig. 3). The anterior pituitary progressively decreases in signal intensity on T1-weighted images with signal drop to near isointensity with the posterior pons by 6 weeks of age [1, 2]. The posterior pituitary gland retains its high signal intensity on T1-weighted images throughout adulthood, with several papers supporting that this high signal results from neurosecretory granules or the protein neurophysin [3–5]. The normal gland enhances symmetrically after intravenous gadolinium with the anterior gland enhancement appearing more noticeable given the posterior gland is already high signal at baseline.

At birth, the gland is physiologically enlarged with a corresponding concave superior margin, flattening with age. The gland is traditionally symmetric in size; however there can be asymmetry in normally functioning glands. In preterm infants, the gland is taller than in normal-term infants, even when correcting for gestational age. This is thought to be the result of reduced insulin-like growth factor 1 and higher levels of growth hormone in premature infants [6]. The pituitary height is fairly stable for the first 2 years of life in normal-term children. Gender-associated differences in the rate of gland growth tend to occur after 5 years of age, with linear growth of the gland up to that point in both boys and girls [7]. Typically, the normal pituitary measures between 2 to 6 mm in height during childhood and up to 10 mm or slightly higher at puberty. In adults, the female glands are slightly larger than their male counter parts [1]. Standards for assessing the pituitary stalk size are not well established, and are based on experience rather than age-appropriate measurements. The stalk is best visualised on post-contrast T1-weighted images because of the strong enhancement.

## Developmental anomalies of the pituitary gland

### Agenesis of the adenohypophysis (Fig. 4)

**Fig. 4:**
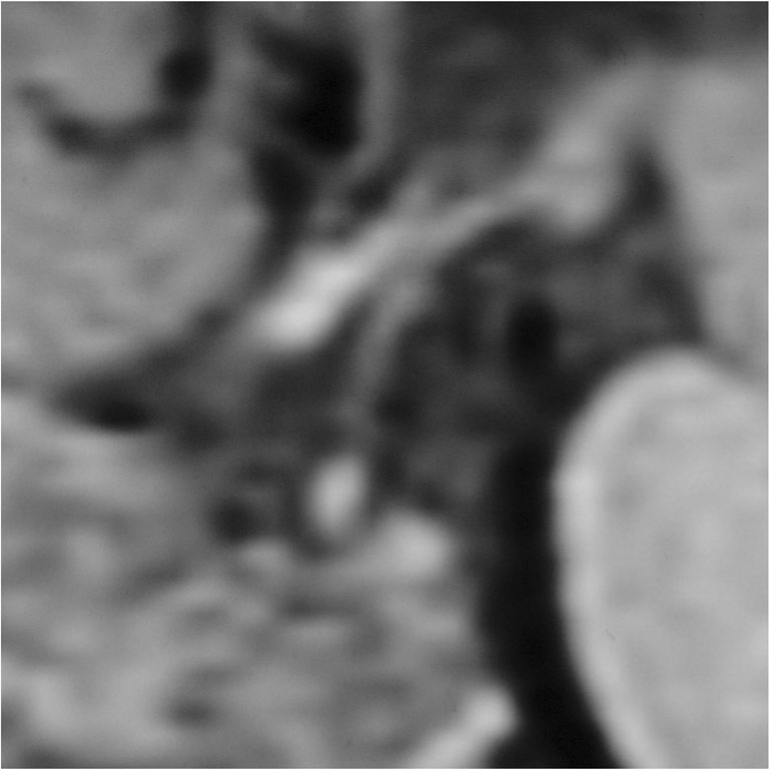
Agenesis of the anterior pituitary gland (adenohypophysis) in an infant with hypopituitarism. Sagittal T1-weighted imaging (T1WI) shows absence of the adenohypophisis. The stalk terminates into a bulbous neurohypophysis with high signal in the dorsal portion of the sella

This is a rare congenital anomaly with a neonatal presentation. The neonates can present with metabolic acidosis, thyroid and adrenal insufficiency, hypoglycaemia, and/or seizures. Associated anomalies of the forebrain and midline craniofacial bones can be present. Newborns are normal in size at birth, which has been explained by the presence of maternal growth hormone. Standard treatment includes replacement of growth hormone, thyroid hormone and glucocorticoids. Magnetic resonance imaging (MRI) findings include the absence of the anterior pituitary gland with an associated shallow sella, and variable length of the infundibulum and position of the posterior pituitary, ranging from the hypothalamus to the sella [8]. The infundibulum may be thinned and terminate into a partially formed bulbous posterior pituitary gland.

### Hypoplasia of the adenohypophysis (Fig. 5)

**Fig. 5:**
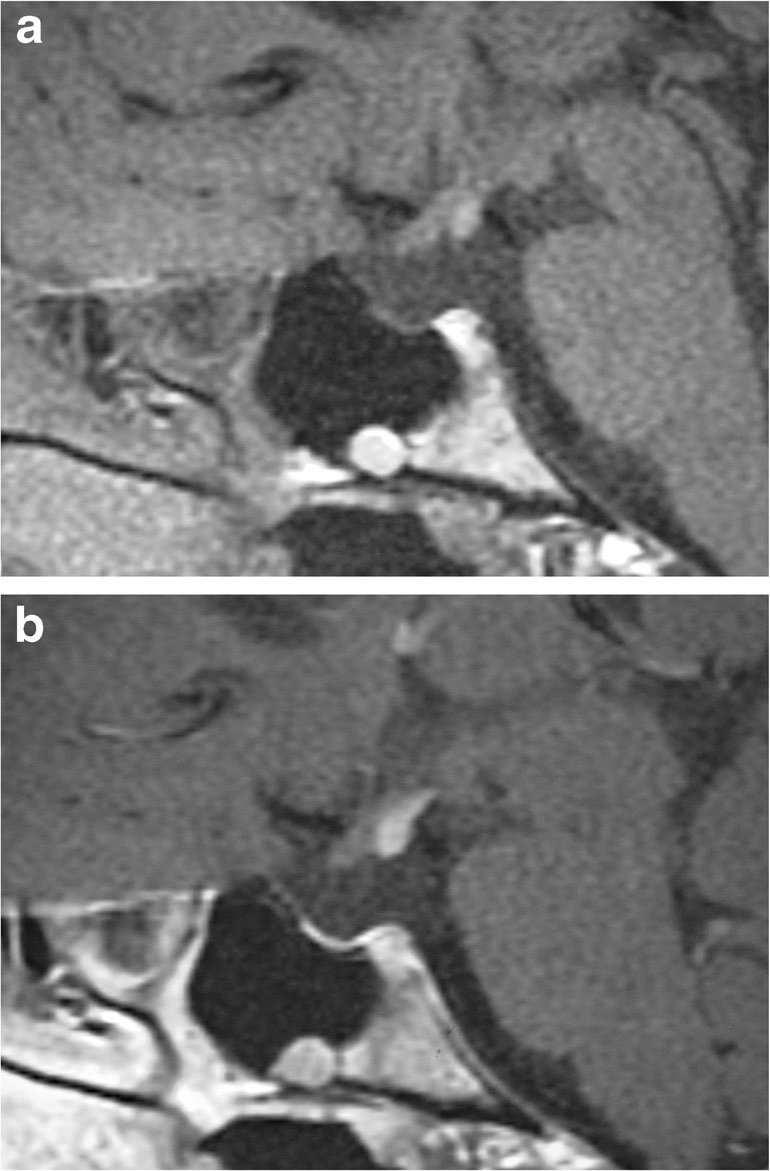
Severe hypoplasia of the anterior pituitary gland. Pre-contrast (**a**) and post-contrast (**b**) MR images show severe hypoplasia of the anterior pituitary gland which shows only minimal contrast enhancement along the floor of the sella. The ectopic posterior pituitary is located adjacent to the optic chiasm

This rare anomaly consists of hypoplasia of the anterior pituitary gland, and also potentially the infundibulum and posterior pituitary gland. It can be associated with varying degrees of endocrine dysfunction, including panhypopituitarism and other congenital anomalies. Some research suggests that traumatic transection of the pituitary stalk during birth or hypoxic injury to the hypothalamus could result in hypoplasia, whereas others suggest an error during embryogenesis [9–12]. MRI findings include varying degrees of hypoplasia of the anterior pituitary gland with variable size and length of the infundibulum. As with agenesis of the adenohypophysis, the posterior pituitary position can range from the hypothalamus to the sella.

### Ectopic posterior pituitary (Fig. 6)

**Fig. 6:**
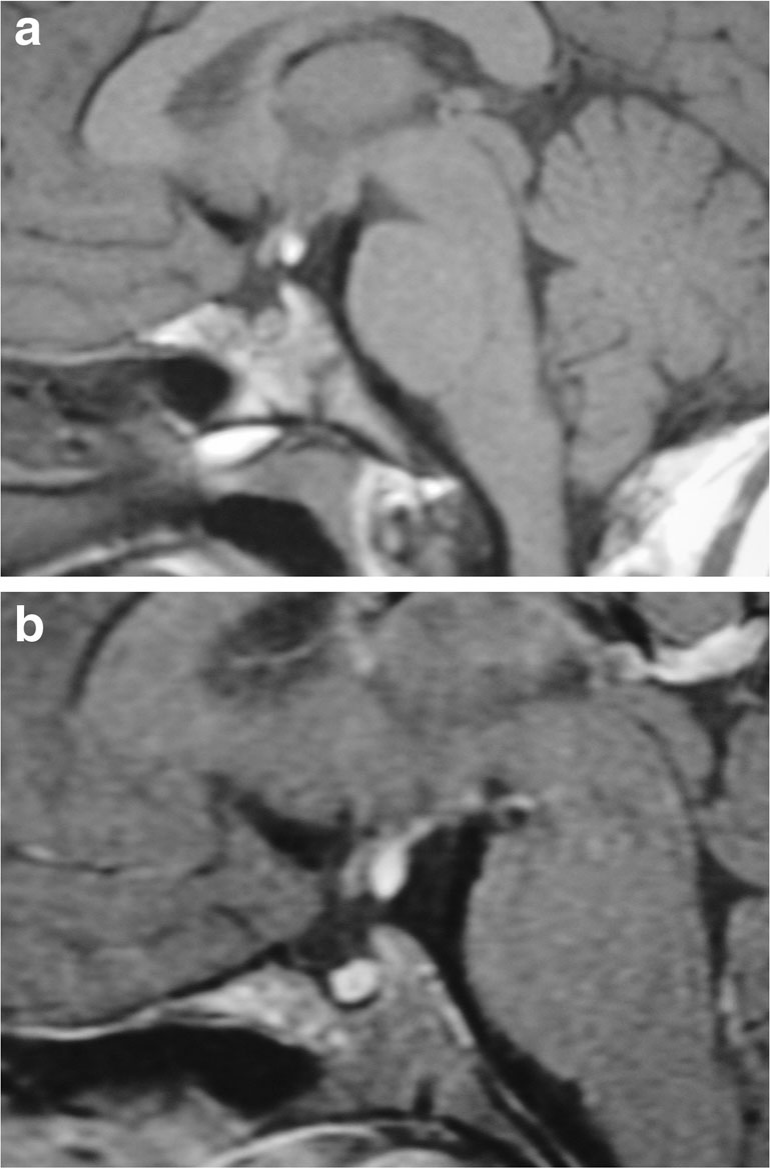
Ectopic posterior pituitary gland. Pre-contrast (**a**) and post-contrast (**b**) MR images show a normal anterior pituitary gland with contrast enhancement and an ectopic posterior pituitary located adjacent to the optic chiasm

This congenital anomaly results in an aberrant position of the neurohypophysis, often located at the undersurface of the hypothalamus. It is associated with pituitary dwarfism, delayed skeletal maturation, Kallman syndrome, septo-optic dysplasia, dysgenesis of the corpus callosum, lobar holoprosencephaly, Chiari 1 malformation or a persistent/enlarged craniopharyngeal canal. It affects males more than females. MRI may show a small anterior pituitary gland with the posterior “bright spot” on T1-weighted imaging (T1WI) located within the upper portion of the infundibulum or the undersurface of the hypothalamus instead of the dorsal portion of the sella [1, 13–15].

### Pituitary duplication (Fig. 7)

**Fig. 7:**
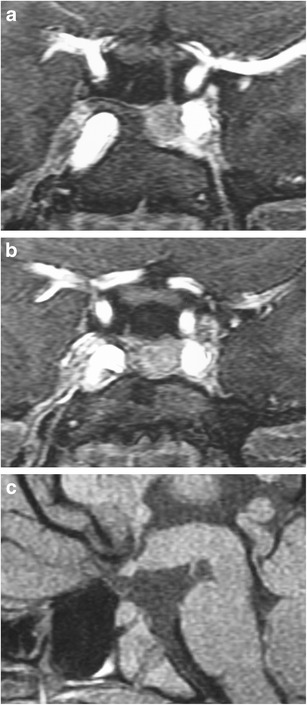
Pituitary duplication. Coronal post-contrast images (**a** and **b**) show two pituitary stalks which extend inferiorly into separate duplicated pituitary glands. Sagittal T1WI (**c**) shows marked thickening of the hypothalamus, referred to as a pseudohamartoma, which has intermediate signal. The pseudohamartoma showed no contrast enhancement (not shown)

This congenital anomaly is related to abnormal separation of the anterior portion of the notochord and prechordal plate during embryogenesis, likely due to teratogenic factors occurring during early embryogenesis [16, 17]. Clinical findings include precocious puberty, facial dysmorphism, hypothyroidism and/or increased prolactin levels. Pituitary duplication may be associated with other anomalies such as dysgenesis of the corpus callosum, absent olfactory bulbs, cerebellar hypoplasia, midline defects, and teratomas. MRI findings include duplicated pituitary stalks and glands within separate pituitary fossa in the sphenoid bone. Abnormal thickening, “pseudohamartoma”, of the hypothalamus with duplication of the infundibular recess of the third ventricle is often seen [16, 18]. Pituitary duplication can also be associated with duplication of the basilar artery [19].

### Hypothalamic hamartoma (Figs. 8 and 9)

**Fig. 8:**
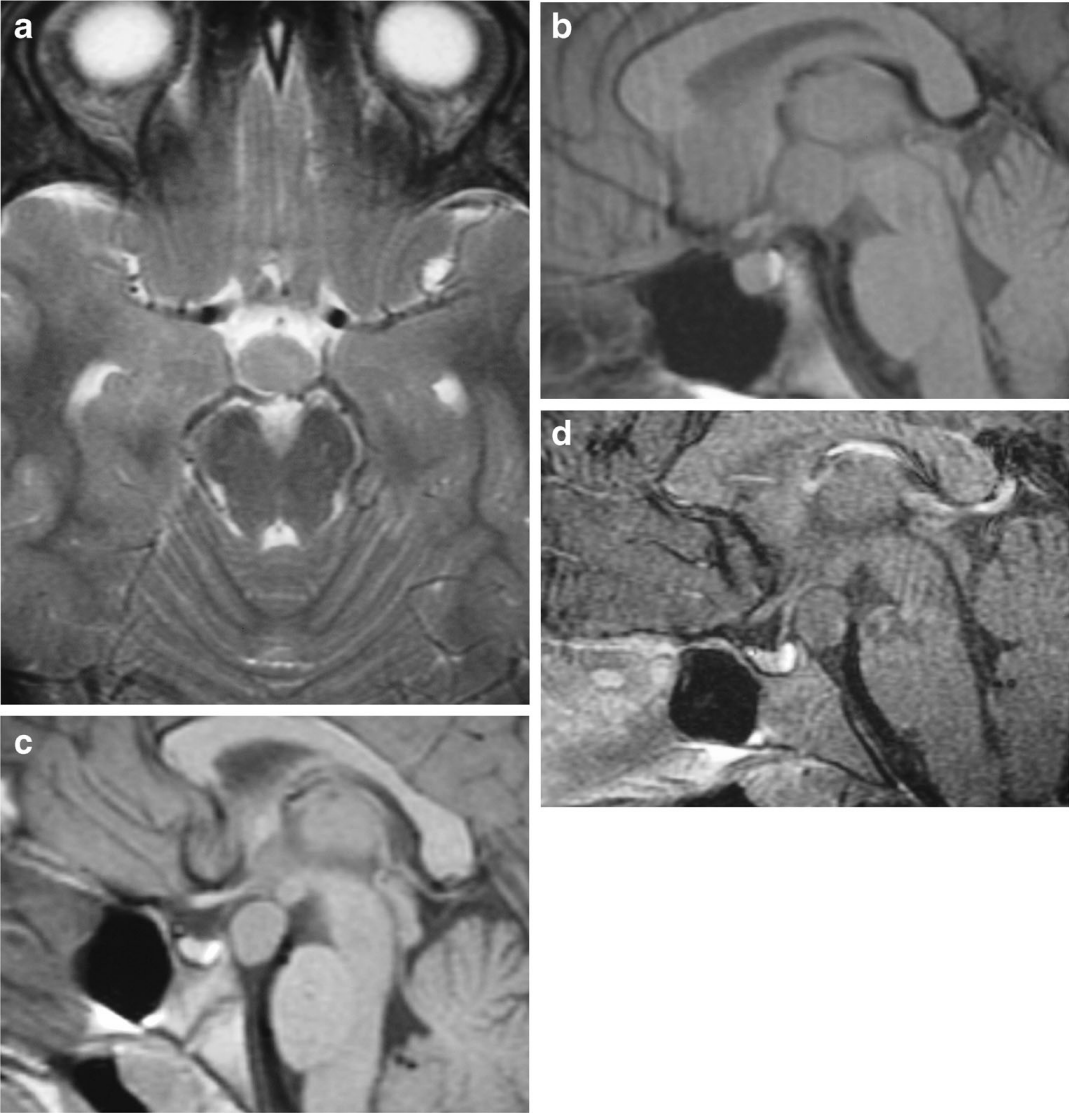
Hypothalamic hamartoma. Axial T2WI (**a**) and sagittal T1WI (**b**) shows a sessile type of hypothalamic hamartoma which has intermediate signal similar to grey matter. Pre-contrast (**c**) and post-contrast (**d**) sagittal T1WI in another patient shows a pedunculated hypothalamic hamartoma with intermediate signal and lacks contrast enhancement

**Fig. 9:**
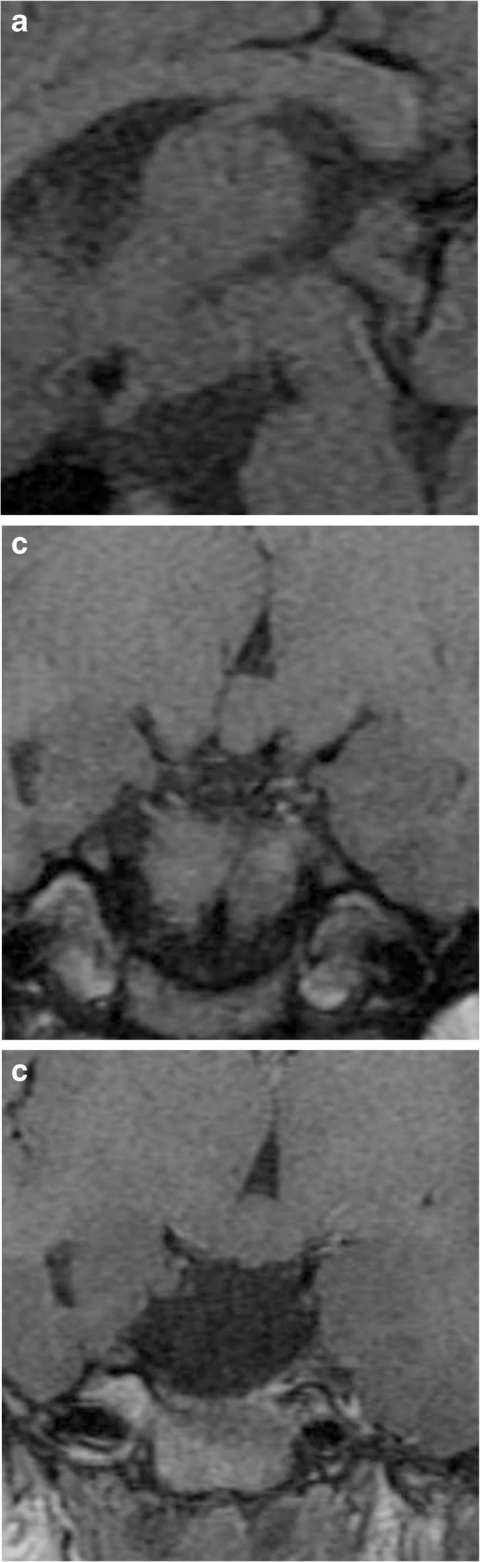
Tuber cinerum hamartoma. Sagittal (**a**) and coronal (**b**) T1WI, and coronal post-contrast T1WI (**c**) show a non-enhancing bulky soft tissue mass in the region of the of the tuber cinerum

Congenital developmental non-neoplastic grey matter heterotopia involving the tuber cinereum, inferior hypothalamus and/or mammillary bodies which are composed of small neuronal cells within a neutrophil-like stroma and scattered fibrillary astrocytes. These lesions typically occur in children with isosexual precocious puberty (0-8 years) or seizures, both gelastic or partial complex, in the second decade [20, 21]. They can be associated with holoprosencephaly, midline facial, cardiac and renal anomalies. MRI demonstrates sessile or pedunculated lesions at the tuber cinereum of the hypothalamus, often intermediate signal on T1WI and T2WI similar to grey matter. Lesions occasionally have slightly high signal on T2WI, with no contrast enhancement or restricted diffusion. Rarely contain cystic and/or fatty portions may be seen in a small minority of cases. The absence of long-term change in size or signal intensity supports the diagnosis [22]. Ictal FDG-PET and SPECT show hyperperfusion during seizures. On MR spectroscopy, there may be an elevated myoinositol peak.

### Persistent craniopharyngeal canal (Fig. 10)

**Fig. 10:**
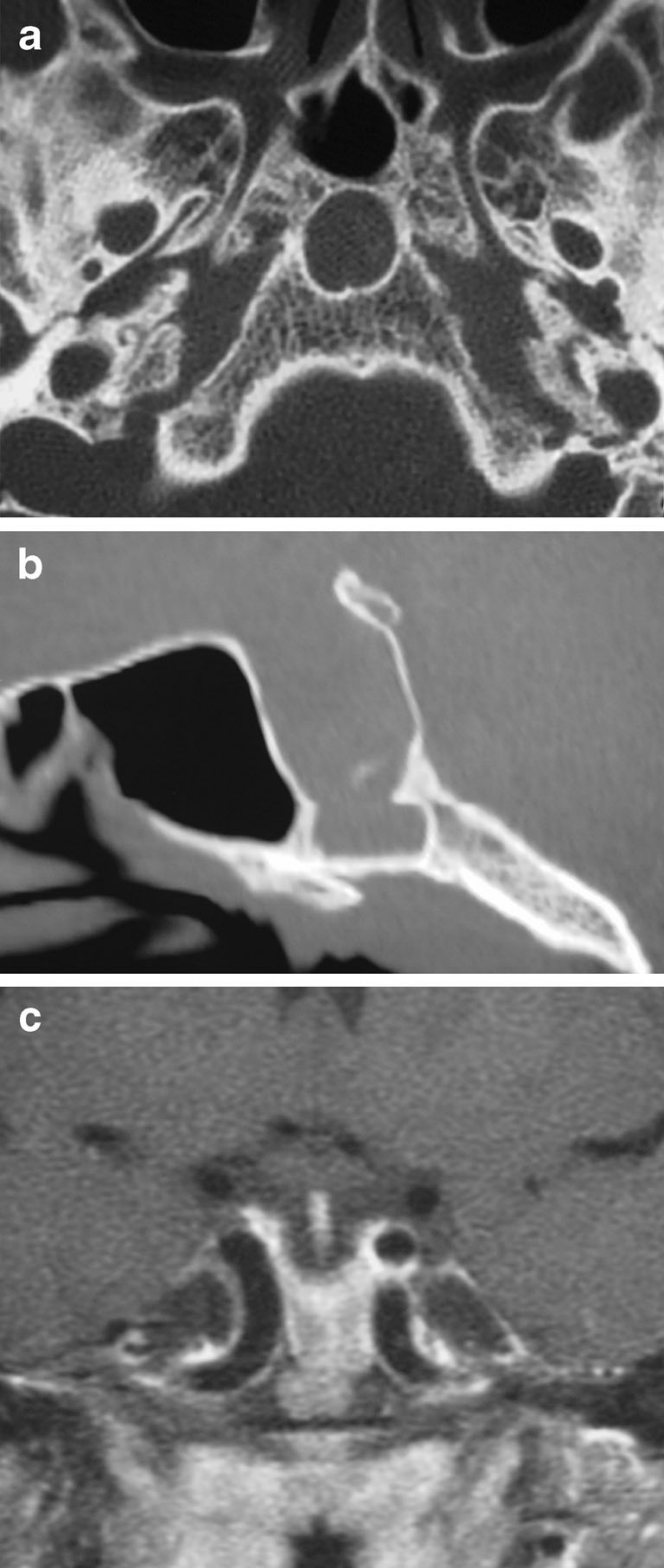
Persistent craniopharyngeal canal. Axial (**a**) and sagittal (**b**) CT shows a markedly enlarged persistent craniopharyngeal canal. Coronal fat-suppressed T1WI shows inferior extension of enhancing pituitary tissue within the abnormally enlarged craniopharyngeal canal (**c**)

Lack of involution of the craniopharyngeal canal, which can contain pituitary tissue. The adenohypophysis develops from evagination of ectodermal cells at the upper posterior portion of the primitive oropharynx, Rathke’s pouch, at 4-5 weeks of gestation. Rathke’s pouch passes between the developing presphenoid and basisphenoid chondrification centres at the upper clivus and sella, where it associates with the descending neuroectoderm of the infundibulum and will go on to form the neurohypophysis. The pathway of Rathke’s pouch is the craniopharyngeal canal, which progressively involutes by 6-7 weeks of gestation. The persistent craniopharyngeal canal on MRI can be associated with trans-sphenoidal meningoencephaloceles, ectopic pituitary tissue, sphenoid teratoma and infrasellar craniopharyngiomas [23, 24]. On MRI, the persistent canal has low T1 signal intensity compared with the high T1 signal of the clivus [13].

### Rathke’s cleft cyst (Fig. 11)

**Fig. 11:**
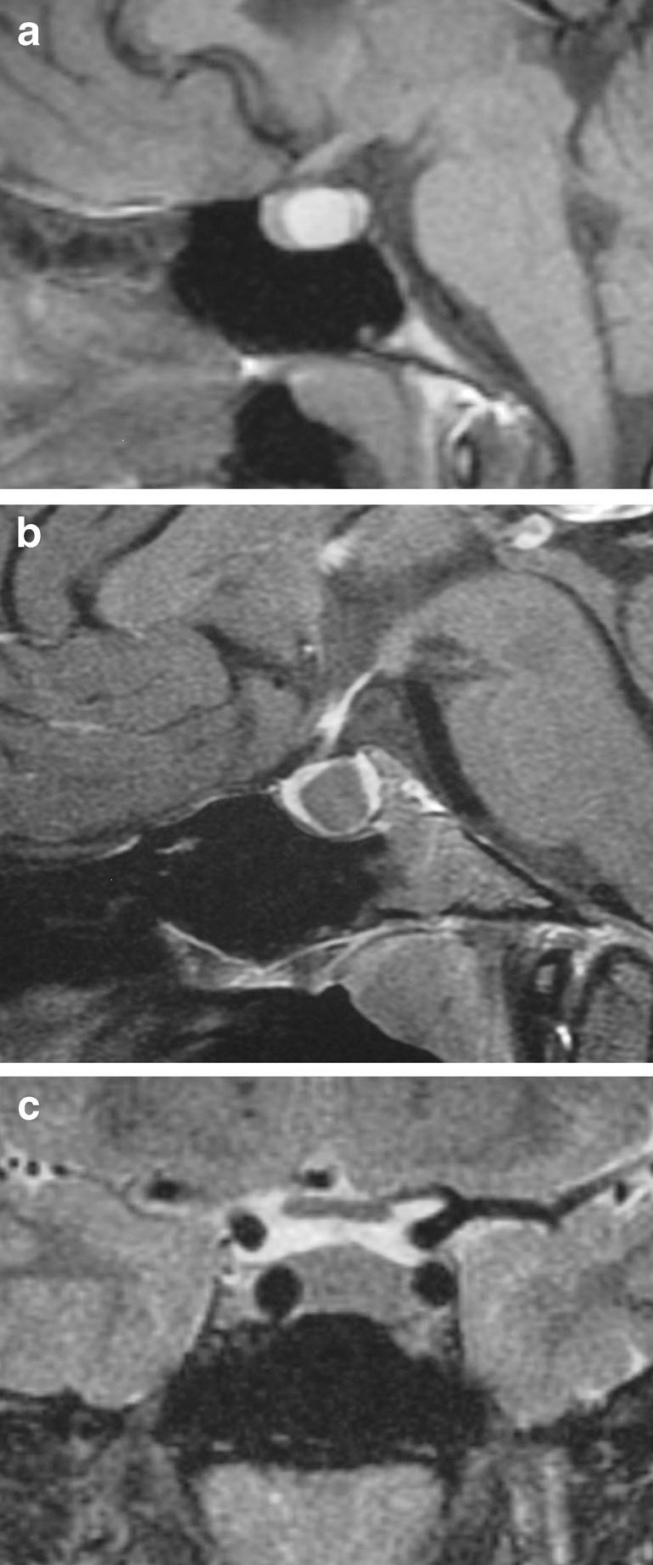
Rathke’s cleft cyst. Sagittal T1WI (**a**) shows a lesion in the central portion of the pituitary gland which has high signal. Post-contrast sagittal fat-suppressed T1WI (**b**) shows lack of enhancement of the Rathke cleft cyst. The cyst has intermediate signal on T2WI (**c**) secondary to slightly elevated protein content

**Fig. 12:**
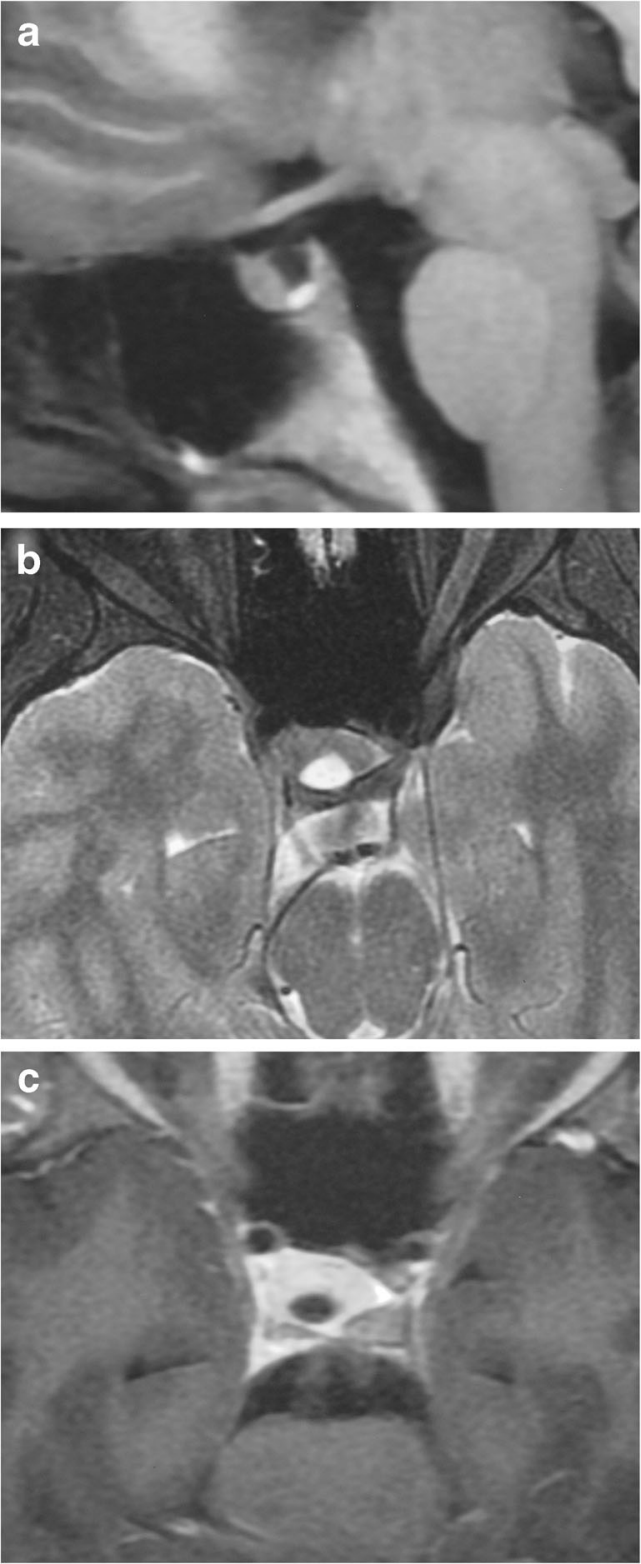
Pars intermedia cyst. Sagittal T1WI (**a**) and axial T2WI (**b**) shows a small cyst between the anterior and posterior pituitary which lacks contrast enhancement on axial fat-suppressed T1WI (**c**)

Benign cystic lesion containing fluid and variable amounts of protein, mucopolysaccharides and/or cholesterol [25, 26]. The cysts arise from epithelial rests of the craniopharyngeal canal. They are 2-3 times more common in women than men, and occasionally become symptomatically related to compression of adjacent structures. The frequency of these cysts on MRI in one study was 1.2 % in childhood [27]. MRI will show a well-circumscribed lesion with variable low, intermediate or high signal on T1WI and T2WI. On T2WI, a small low-signal nodule may be seen within the predominant high signal of the lesion, which does not enhance after contrast administration. The incidence of these intracystic nodules varies from 17 to 78 % [28, 29]. There is no contrast enhancement centrally; however, occasionally there may be a thin peripheral rim of enhancement from associated inflammation. The majority are located in the intrasellar region or combined intrasellar and suprasellar region with reports that only 8.3 % are exclusively suprasellar [30, 31]. Most are stable in size, and some may disappear spontaneously. Pars intermedia cysts (Fig. 12) can also form along the posterior margin of Rathke’s pouch, are typically less than 3 mm, with many considered Rathke’s cleft cysts on imaging as they are difficult to distinguish from one another and Rathke’s cleft cysts are more common [1].

### Cephaloceles (meningoceles or meningoencephaloceles) (Fig. 13)

**Fig. 13:**
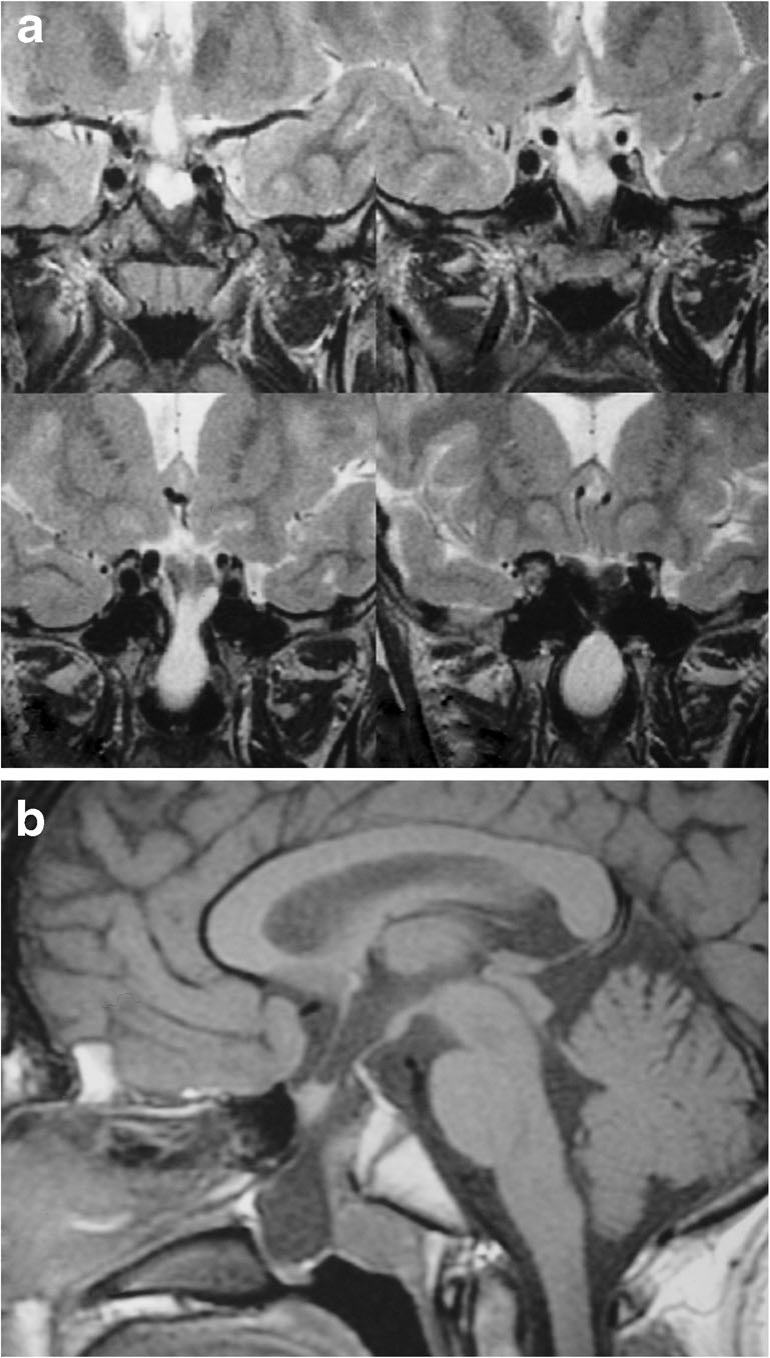
Cephalocele in a 17-year-old girl. Coronal T2WI (**a**) and sagittal T1WI (**b**) shows a meningocele extending inferiorly into the nasopharynx through an osseous defect in the sphenoid bone posterior to the pituitary gland

Congenital malformation involving the lack of separation of neuroectoderm from surface ectoderm with resultant localised failure of bone formation. There is a wide range of abnormalities which affect the skull base within the sella turcica, ranging from a persistent craniopharyngeal canal to trans-sphenoidal and sphenoethmoidal cephaloceles, many presentations of which are extremely rare. For example, the reported incidence of trans-sphenoidal meningoencephaloceles is 1 per 700,000 live births [13, 32]. Clinical findings include difficulty in feeding and nasal obstruction in the first year, and potential for cerebrospinal fluid (CSF) leaks and meningitis. MRI will show a defect in the skull, through which there is either herniation of meninges and CSF (meningocele), or meninges, CSF and brain tissue (meningoencephaloceles).

### Epidermoid (congenital cholesteatoma) (Fig. 14)

**Fig. 14:**
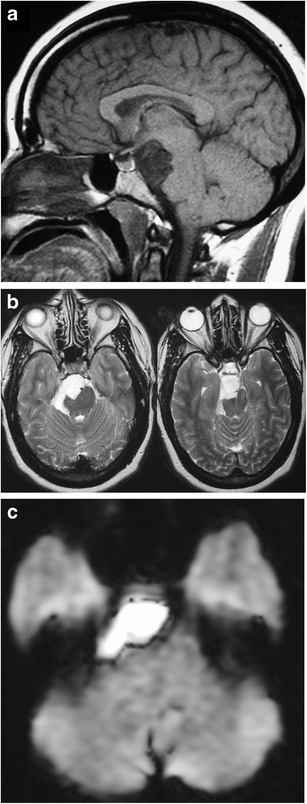
Epidermoid. Sagittal T1WI (**a**) shows an extra-axial lesion along the endocranial surface which has heterogeneous mostly low-intermdiate signal, and on T2WI has high signal (**b**). Diffusion-weighted imaging (DWI) (**c**) shows restricted diffusion

Non-neoplastic congenital or acquired extra-axial off-midline lesion filled with desquamated cells and keratinaceous debris, usually with mild mass effect on the adjacent brain. Epidermoids grow by accumulation of desquamated epithelial cells into the centre of the cyst [33–35]. MRI will show a well circumscribed spheroid or multilobulated extra-axial ectodermal inclusion cystic lesion with low-intermediate signal on T1WI, high signal on T2WI and no contrast enhancement. They can be distinguished from arachnoid cysts, as they are typically high signal on FLAIR and DWI sequences, with corresponding low signal on ADC [36].

### Dermoid (Fig. 15)

**Fig. 15:**
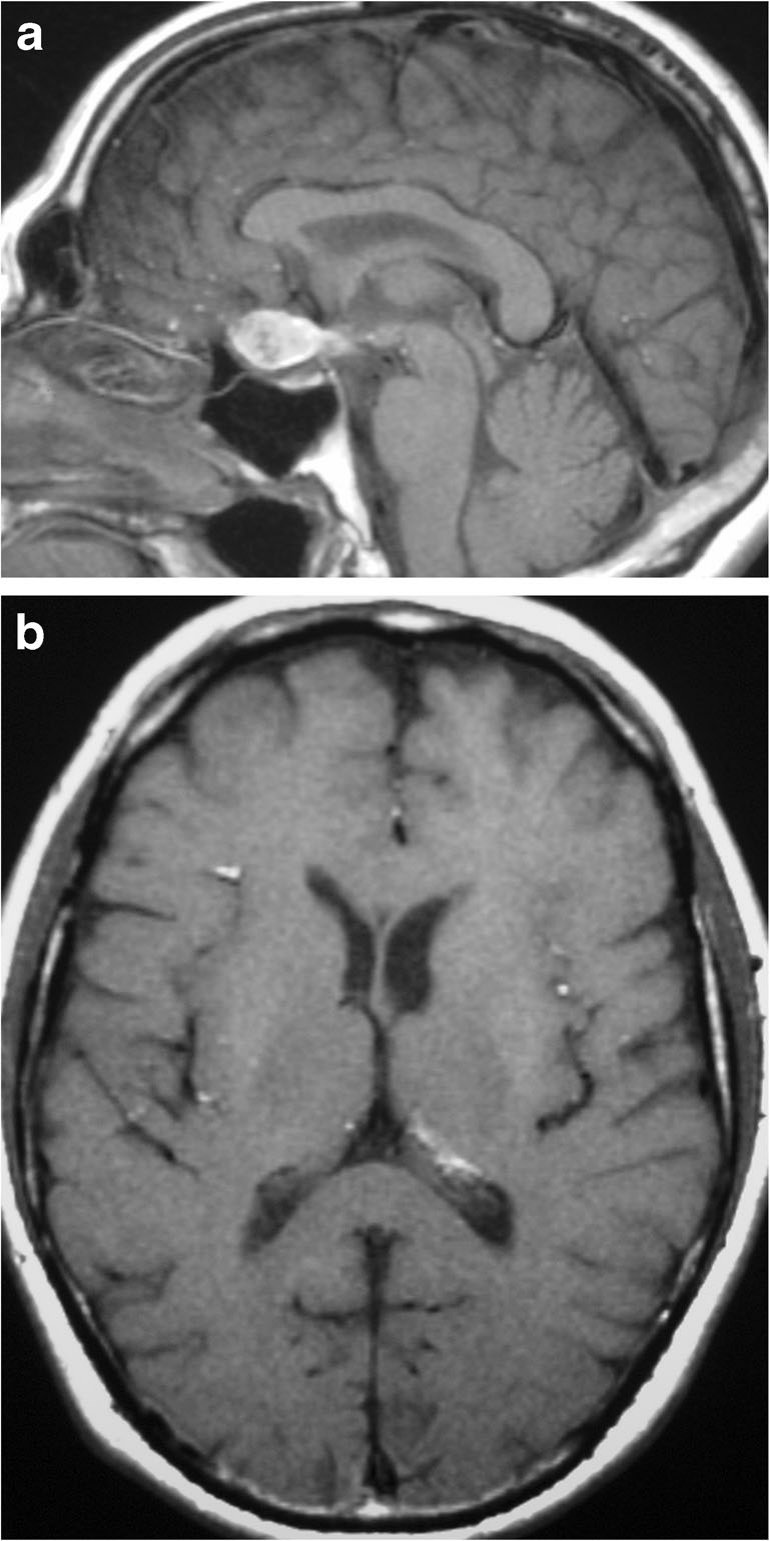
Dermoid. Sagittal (**a**) and axial (**b**) T1WI shows an extra-axial lesion in the suprasellar cistern which has high signal. Multiple small foci with high signal are seen in the sulci and within the left lateral ventricle representing ruptured dermoid contents in the leptomeninges

Non-neoplastic congenital or acquired ectodermal inclusion cystic lesion, which like an epidermoid, is lined by squamous epithelium, and are filled with lipid material, cholesterol, desquamated cells and keratinaceous debris. They are distinguished from epidermoids by containing deeper dermal elements and commonly arise from the midline. They typically present in the 20- to 30-year age range, but can also be seen in adolescence, with males slightly more affected than females [36]. MRI will show a well-circumscribed spheroid or multilobulated extra-axial lesion, usually with high signal on T1WI, variable signal on T2WI and no contrast enhancement. Fluid/fluid or fluid/debris levels may be present. They can cause a chemical meningitis if the dermoid cyst ruptures into the subarachnoid space [37, 38].

### Arachnoid cyst (Fig. 16)

**Fig. 16:**
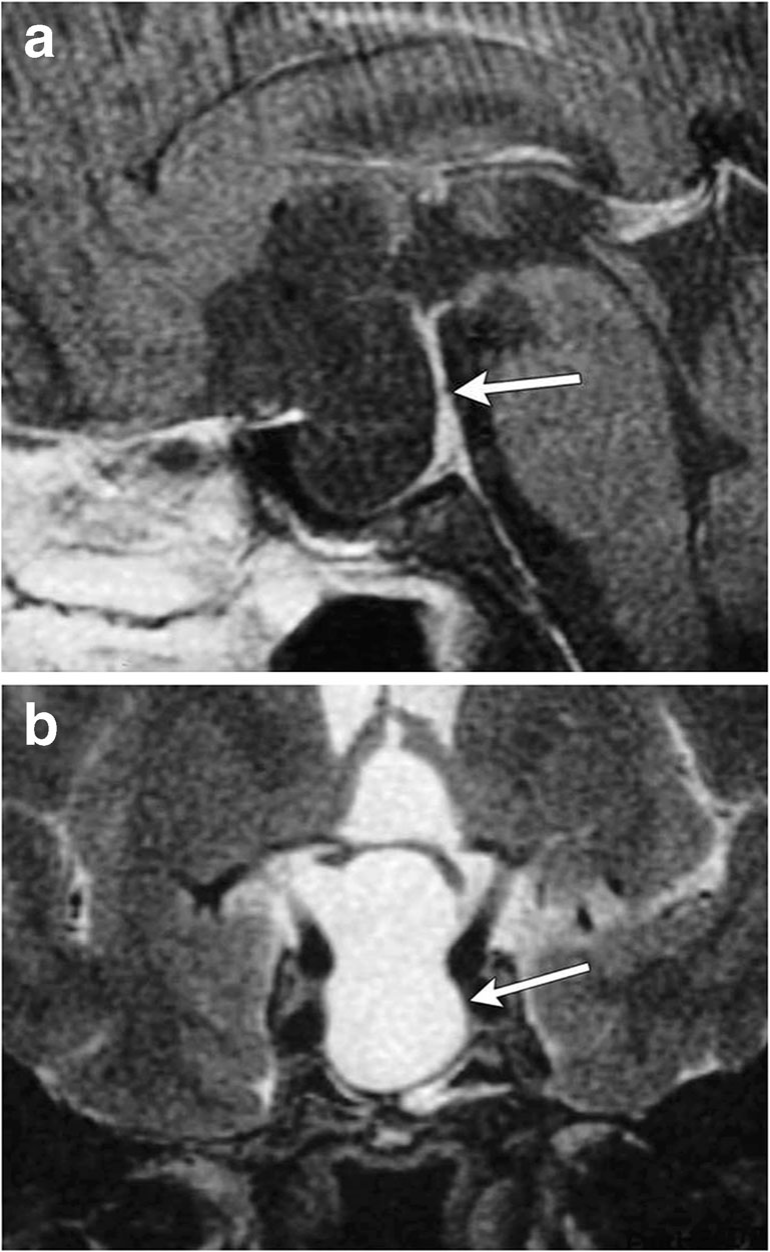
Arachnoid cyst. Sagittal T1WI (**a**) and coronal T2WI (**b**) show a well defined cystic mass with low T1 and high T2 signal intensity with sellar and suprasellar components

Congenital arachnoid cysts are found in 0.16 % of all newborns with approximately 9-15 % occurring in the sellar region, with suprasellar cysts more commonly seen than intrasellar cysts. Most cases are asymptomatic; however, they may cause headache, optic nerve compression, psychomotor retardation, endocrine dysfunction or hydrocephalus [39, 40]. Secondary arachnoid cysts may result from trauma, haemorrhage or infection [41]. On imaging, the cysts have low signal intensity on T1WI, FLAIR and DWI, high signal intensity on T2WI, no contrast enhancement, and may cause mild mass effect on adjacent brain.

### Lipoma (Fig. 17)

**Fig. 17:**
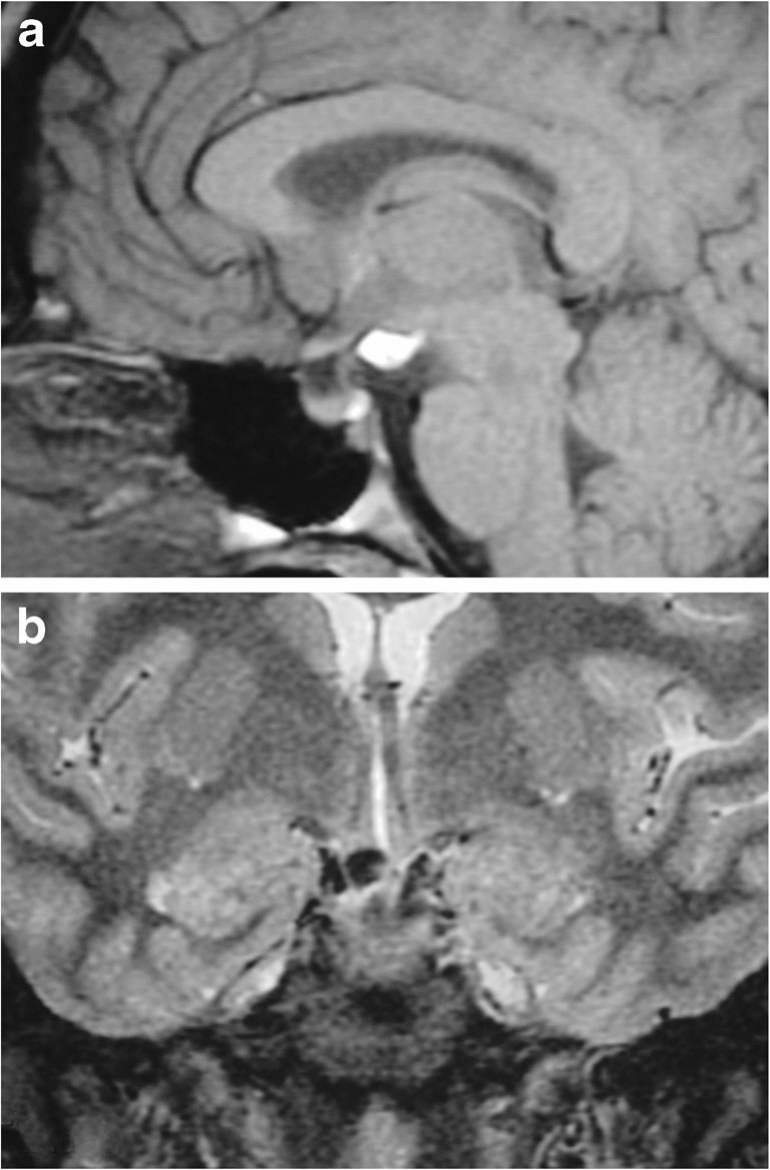
Lipoma. Sagittal T1WI (**a**) shows a lipoma with high signal along the inferior surface of the hypothalamus. The signal on the lipoma is nulled on coronal fat-suppressed T2WI (**b**)

Benign fatty lesions resulting from a congenital malformation, often located at or near the midline. In the sellar region, they occur along the surface of the infundibulum, floor of the third ventricle, or adjacent to cranial nerves, and are usually discovered incidentally, unless they cause a mass effect [42, 43]. They may contain calcifications and/or traverse blood vessels. MRI signal is isointense to subcutaneous fat on all sequences, with fat suppressed images useful in distinguishing from haemorrhagic or proteinaceous lesions.

### Empty sella (Fig. 18)

**Fig. 18:**
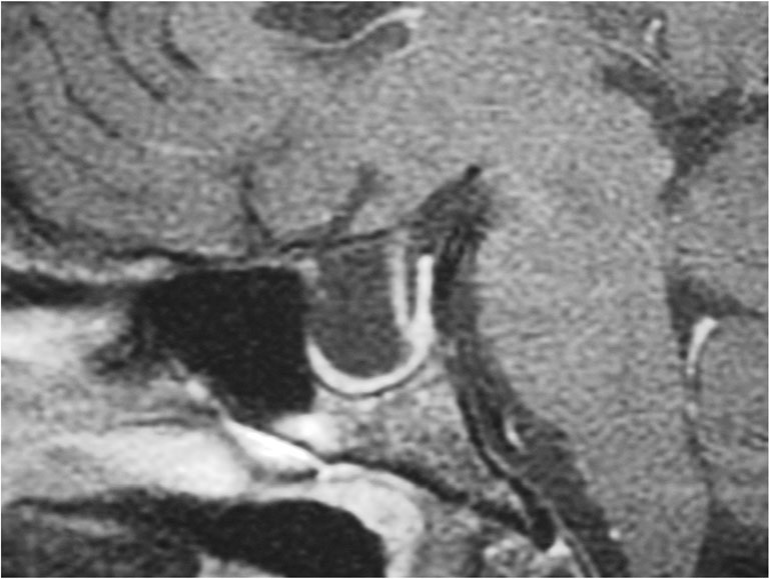
Empty sella. Sagittal post-contrast T1WI with fat-suppression show an enlarged sella mostly filled with CSF. The enhancing flattened pituitary tissue is located along the floor of the sella

Common incidental finding involving the pituitary gland and sella, which can result from a weakened or fenestrated diaphragma sella allowing CSF pulsations to flatten the pituitary gland. They are associated with elevated intracranial pressure, a posteriorly placed optic chiasm, and are considered a normal variant or termed “primary empty sella turcica” in the absence of surgery, radiation therapy or medically treated intrasellar tumour [44]. In children, the incidence of an empty sella is seen more often in boys and is more likely to be associated with clinical symptoms and endocrinopathies, particularly growth hormone or multiple pituitary hormone deficiencies [45]. MRI findings include a thinned/flattened pituitary gland along the floor of the sella, with the infundibulum in its normal midline position. The flattened pituitary gland has normal MRI signal characteristics and contrast enhancement.

## Pituitary lesions

### Pituitary adenoma (Fig. 19)

**Fig. 19:**
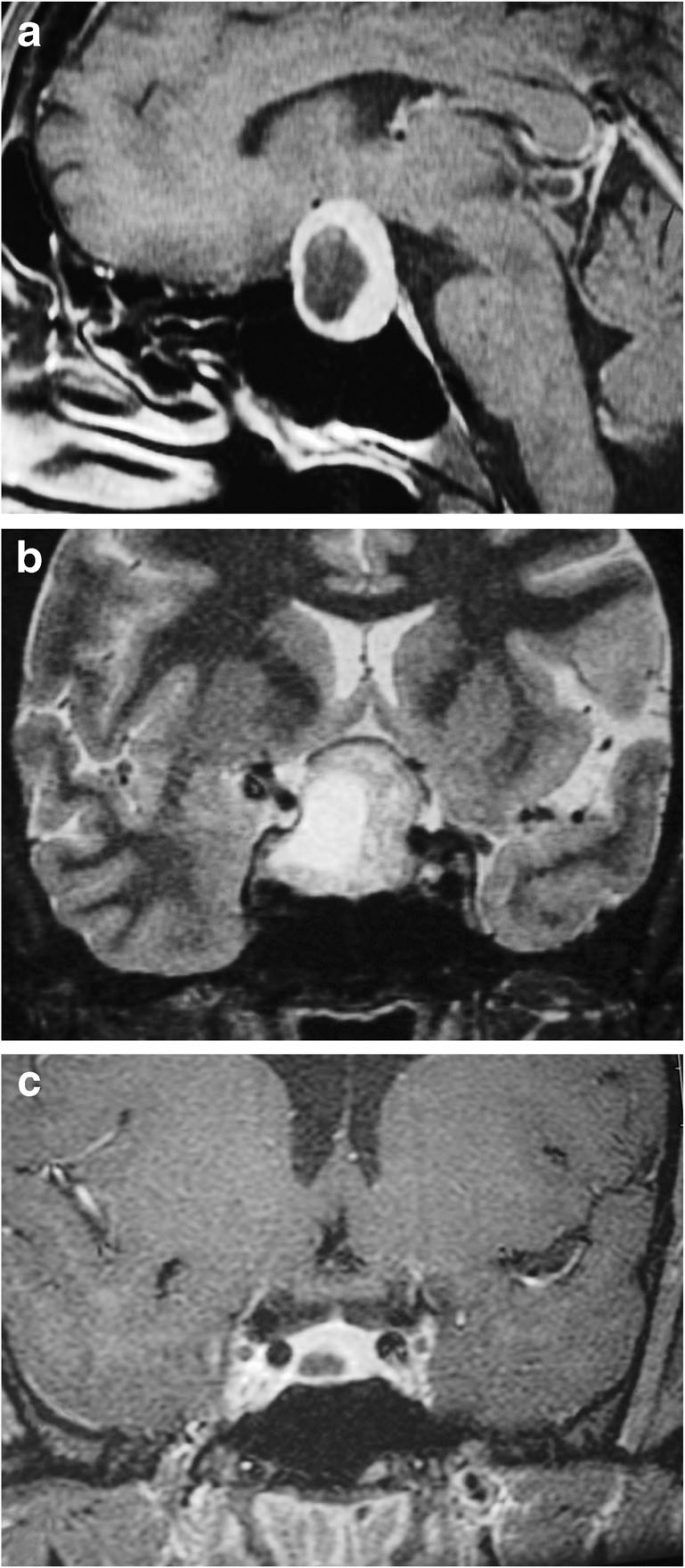
Pituitary adenomas. Sagittal post-contrast fat-suppressed T1WI (**a**) shows an heterogenously enhancing pituitary macroadenoma which contains a non-enhancing portion with high signal on T2WI (**b**). secondary to cystic degeneration. Coronal post-contrast fat-suppressed T1WI (**c**) shows a pituitary microadenoma which shows less early contrast enhancement than the normal pituitary tissue

In comparison to adults, pituitary adenomas are rare in the paediatric population, accounting for approximately 2.7 % of all supratentorial tumours. Most paediatric adenomas are functioning, with only 3-6 % non-functioning, compared to one-third of non-functioning adenomas in adults [46–48]. Microadenomas may be associated with endocrine abnormalities related to over-secretion of hormones, with prolactinomas being the most common. Rarely, extensive haemorrhage can occur involving the adenoma, resulting in pituitary apoplexy (Sheehan syndrome). Macroadenomas with suprasellar extension can cause compression and displacement of the optic chiasm with associated visual disturbances (bitemporal hemianiopsia). Most pituitary adenomas arise from sporadic mutations; however, 5 % can be associated with inherited disorders such as McCune Albright syndrome, Carney complex and multiple endocrine neoplasia type 1 (MEN 1).

MRI findings of a microadenoma (<10 mm) commonly have intermediate signal on T1WI and T2WI, and may show characteristics of cysts, haemorrhage or necrosis. They typically show less contrast enhancement than normal pituitary tissue, often best visualised with dynamic early-phase imaging. In macroadenomas (>10 mm), they commonly have intermediate signal on T1WI and T2WI similar to grey matter, and may also have cystic, haemorrhagic or necrotic components. They can extend into the suprasellar cistern with a waist at the diaphragm sella or into the cavernous sinus, with occasional invasion into the skull base.

## Juxtasellar lesions

### Craniopharyngioma (Fig. 20)

**Fig. 20:**
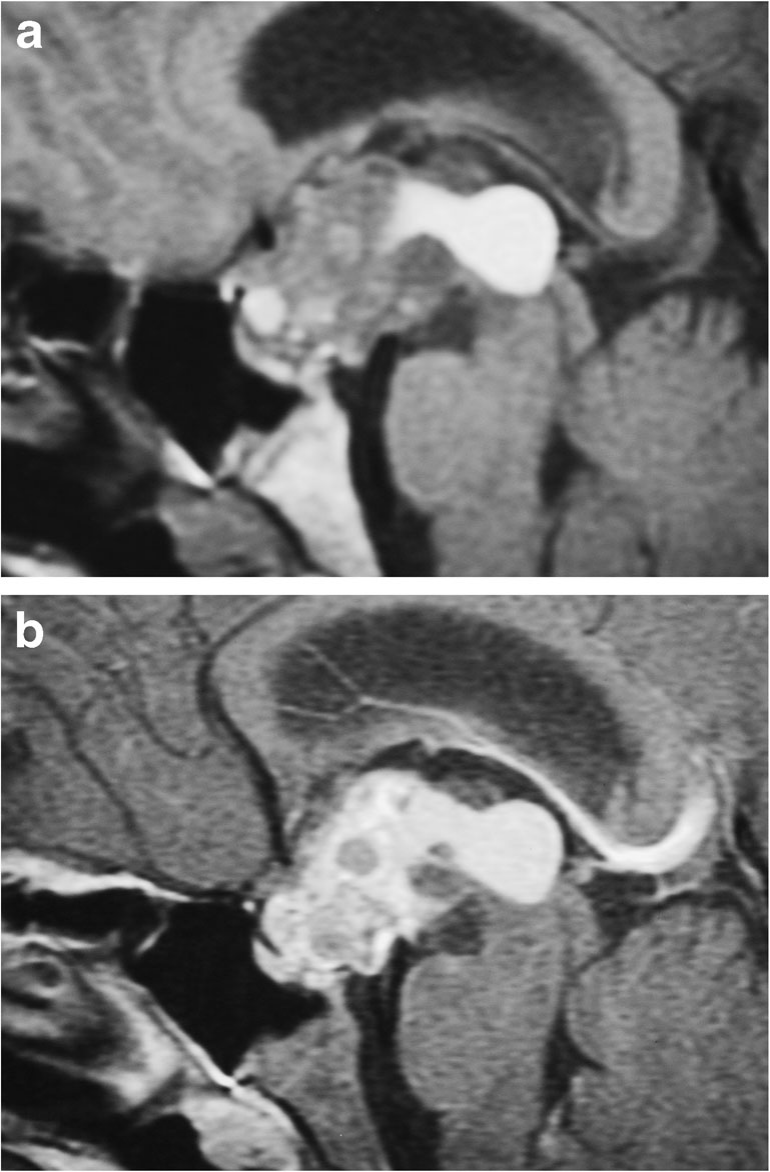
Craniopharyngioma. Sagittal T1WI (**a**) shows a lobulated lesion filling the suprasellar cistern which contains zones of low, intermediate and high signal. Sagittal post-contrast T1WI (**b**) shows the lesion to have heterogeneous contrast enhancement

Usually histologically benign locally aggressive lesions which arise from squamous epithelial rests along Rathke’s cleft, occurring in a bimodal age distribution, in children (<10 years) and adults (>40 years). Craniopharyngioma’s account for 3 % of all intracranial tumours. They are categorised into adamantinomatous and squamous-papillary types. The adamantinomatous type is more common and has a bimodal age distribution occurring in children and adults, whereas the squamous-papillary type usually occurs in adults. Although histologically benign, their insinuating pattern of growth makes complete surgical excision very difficult and not often possible.

The adamantinomatous type usually demonstrates circumscribed lobulated margins on MRI, with the suprasellar location seen at a greater frequency than intrasellar. They show variable low, intermediate and/or high signal on T1WI and T2WI, with or without a nodular or rim of contrast enhancement. They may contain cysts, lipid components and calcifications. In children, the cystic parts tend to predominate, and frequently contain highly proteinaceous fluid, which may be bright on T1WI [49].

### Glioma (Fig. 21)

**Fig. 21:**
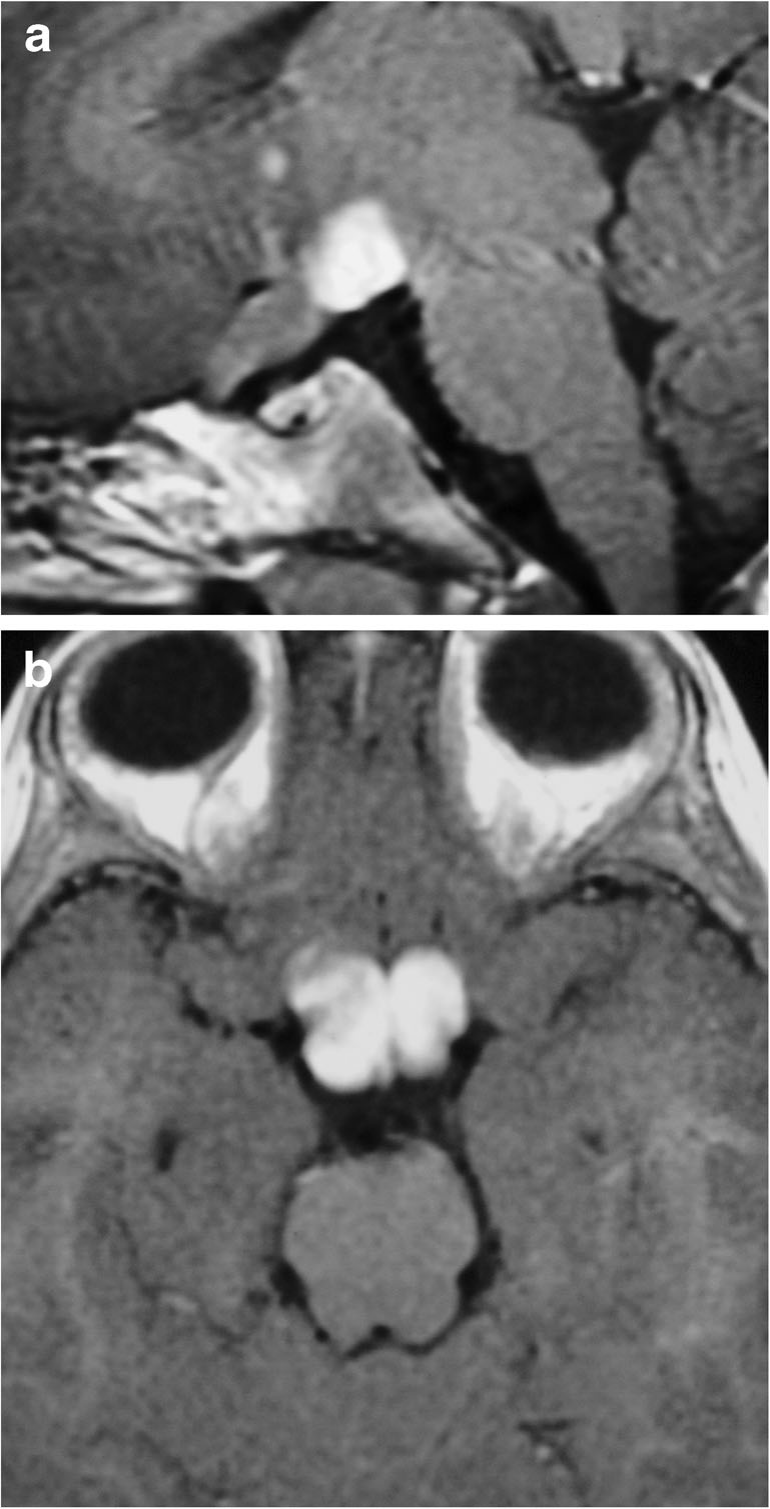
Glioma in a 2-year-old girl with neurofibromatosis type 1. Sagittal (**a**) and axial (**b**) post contrast T1WI shows a partially enhancing lesion enlarging the optic chiasm and proximal optic nerves

Lesions which may occur anywhere along the optic pathway and account for 4-6 % of brain tumours in children, with a median age of diagnosis from 5-9 years. Approximately half of patients with optic pathway gliomas have neurofibromatosis type 1 (NF1), with symptomatic lesions only occurring in 1-5 % of patients [50]. Most tumours are slow growing WHO grade I-II astrocytomas, often the pilocytic type, however tumours may show a wide spectrum of progression. On MRI, there is fusiform and/or nodular enlargement of the optic chiasm and/or optic nerves with thickening of the third ventricular floor and hypothalamus. They usually have low-intermediate signal on T1WI, intermediate-high signal on T2WI and variable contrast enhancement. Larger lesions may have cystic components and grow directly into the pituitary stalk [1].

### Pilomyxoid astrocytoma (Fig. 22)

**Fig. 22:**
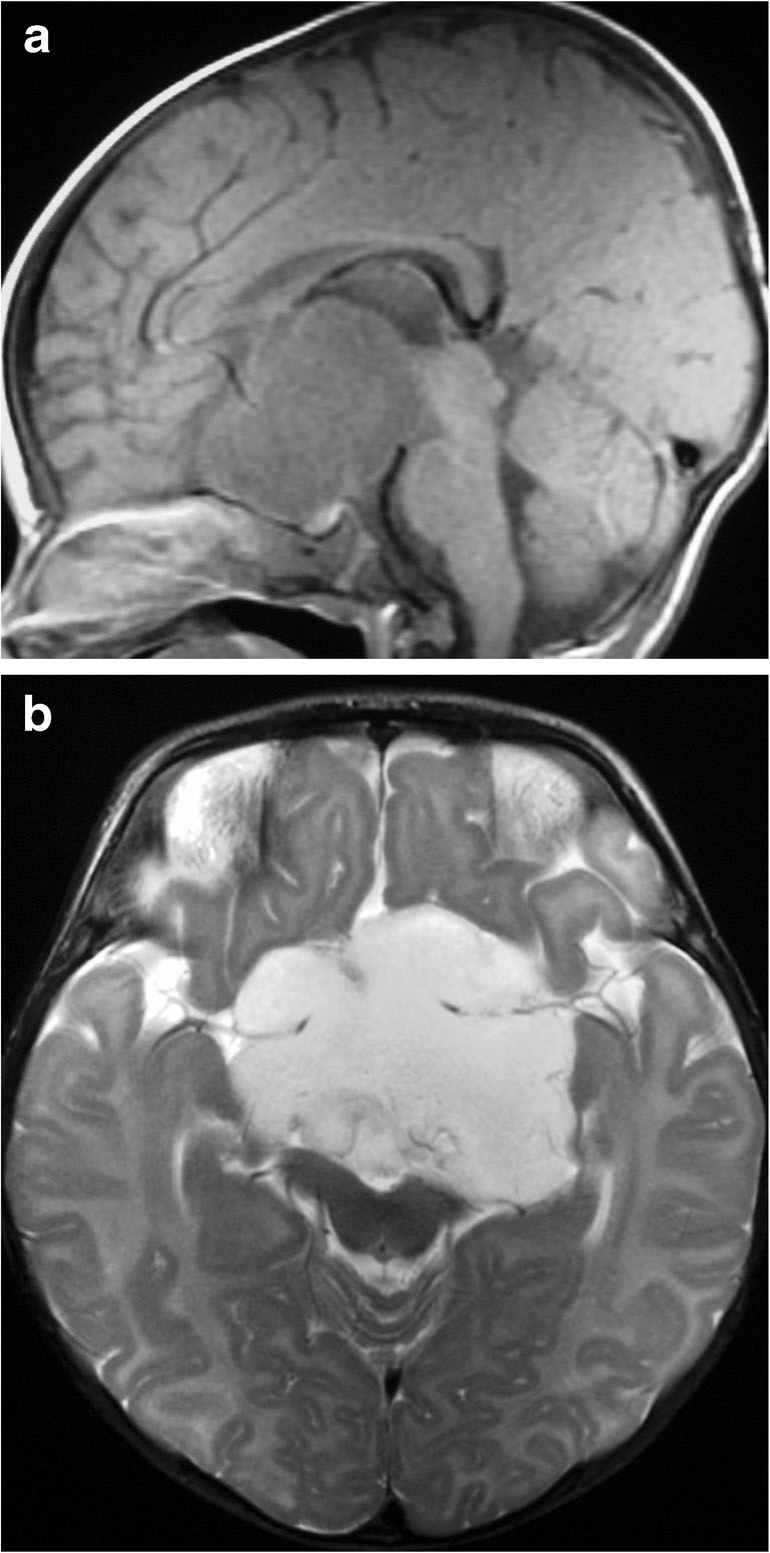
Pilomyxoid astrocytoma in a 4-month-old baby girl. Sagittal T1WI (**a**) shows a large hypothalamic lesion with intermediate signal which extends superiorly into and filling the third ventricle, and inferiorly into the suparsellar cistern. The tumour has mostly high signal on axial T2WI (**b**)

Rare neoplasms (WHO grade II) which contain prominent mucoid matrix and angiocentric pattern of bipolar neoplastic astrocytes. Typically, they occur in children in the first and second decades, with a mean age of 7 years [51]. They tend to occur at a younger age and are more aggressive with a higher rate of local recurrence than pilocytic astromcytomas. They have a strong predilection for the hypothalamus and suprasellar cistern [52]; however, they can also occur in the thalamus, cerebellum, brainstem, temporal lobe and spinal cord. The presence of intra-tumoural haemorrhage is the most prominent imaging characteristic to suggest a pilomyxoid astrocytoma over a pilocytic astrocytoma, 25 % versus 8 %, respectively [51]. MRI demonstrates a solid/cystic focal lesion with low-intermediate signal on T1WI, high signal on T2WI and FLAIR, variable contrast enhancement and may show increased ADC values.

### Germ cell tumours—germinoma (Fig. 23)

**Fig. 23:**
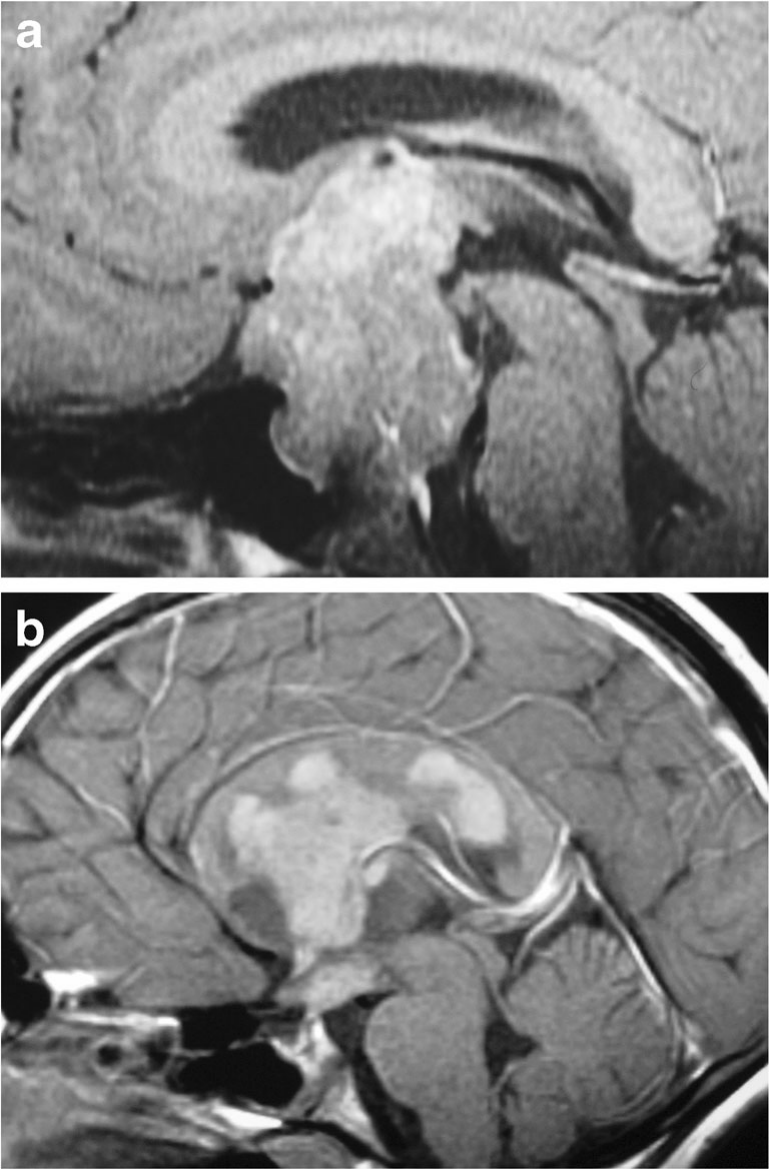
Germ cell tumours in two different children. Post-contrast T1WI (**a**) shows a large enhancing lesion in the suprasellar cistern extending superiorly into the third ventricle and posteriorly into the prepontine cistern. Sagittal post-contrast T1WI (**b**) in another child shows enhancing, disseminated germ-cell tumour in the suprasellar cistern, third and lateral ventricles, with involvement of the corpus callosum and optic chiasm

Extra-gonadal germ cell tumours include: germinoma (most common), mature teratoma, malignant teratoma, yolk sac tumour, embryonal carcinoma and choriocarcinoma. They account for 0.6 % of primary intracranial tumours, with an incidence of 0.09 per 100,000. Peak incidence occurs between 10 and 14 years, with 90 % occurring in patients less than 25 years. They occur more frequently in males. Prognosis depends on the histological subtype, with a 10 year survival rate for germinomas of over 85 %. Other germ cell tumours have a lower survival rate, particularly those containing non-germinomatous malignant cells. Tumours often have intermediate signal on T1WI, slightly high signal on T2WI, and show contrast enhancement. They are typically centred on the pituitary stalk with the posterior pituitary bright spot often absent. They may look similar to Langerhans’ cell histiocytosis (LCH) or lymphocytic hypophysitis [53]. Contrast-enhancing disseminated subarachnoid and/or intraventricular tumour may occur through CSF seeding.

### Germ cell tumours—teratoma (Fig. 24)

**Fig. 24:**
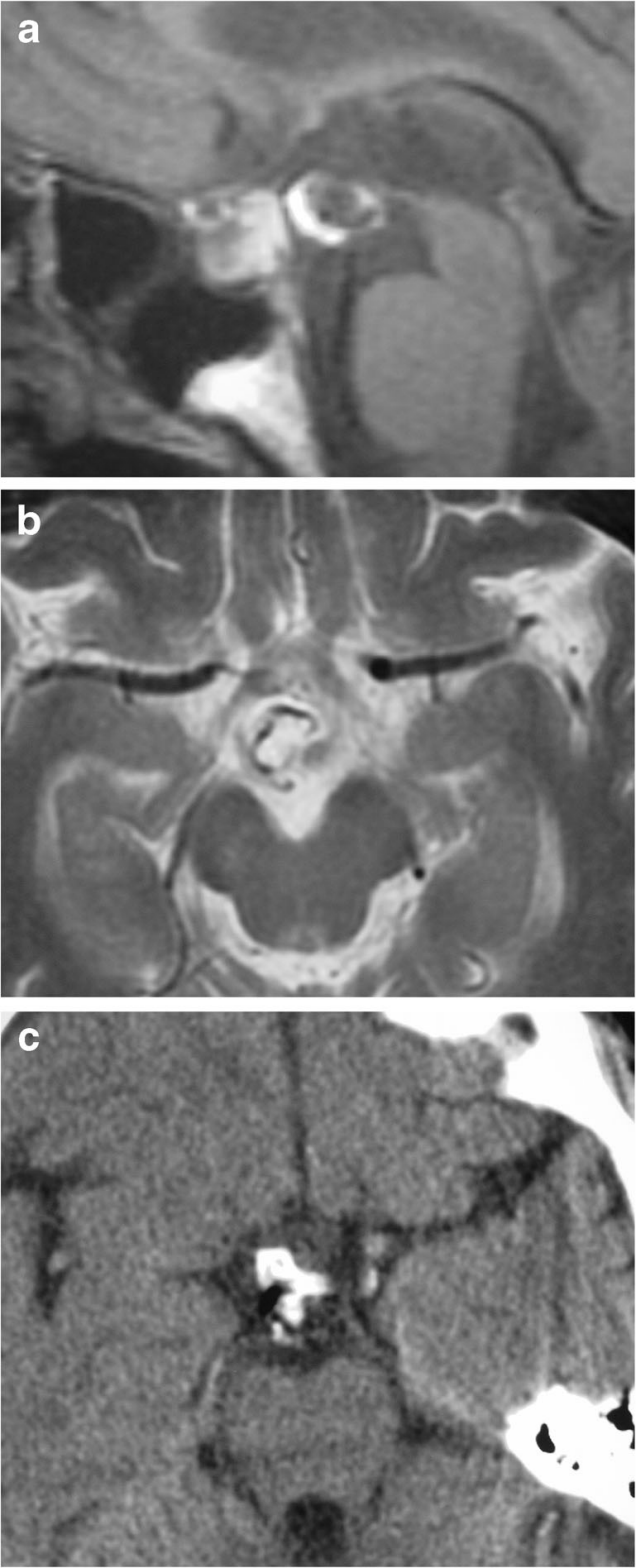
Teratoma. Sagittal T1WI (**a**) and axial T2WI (b) show a lesion at the undersurface of the hypothalamus which has mixed low, intermediate and high signal. Axial CT image (**c**) shows the lesion to contain zones of calcification, fat and intermediate soft-tissue attenuation

The second most common type of germ cell tumour behind a germinoma. They occur in children, with a greater frequency in boys, accounting for 0.5-1.5 % of all childhood brain tumours, and >50 % of intracranial tumours in infants less than 2 months [54, 55]. Histologically, they demonstrate benign or malignant types, composed of derivatives of ectoderm, mesoderm and/or endoderm. On MRI, they are relatively circumscribed lesions, most common in decreasing order frequency within the pineal region, suprasellar region, and third ventricle. On MRI, they demonstrate heterogeneous signal intensity on T1WI, T2WI and post-contrast sequences, with suppression of signal on fat-saturated imaging. Marked enhancement of the solid portions of the tumour is a key feature in distinguishing a mature versus a malignant teratoma, with malignant tumours demonstrating enhancement. Additionally, they typically present without perilesional high T2 signal due to intact capsules and an undamaged blood-brain barrier [55]. They may contain calcifications with low signal on T1WI and T2WI.

### Tuberculous meningitis (Fig. 25)

**Fig. 25:**
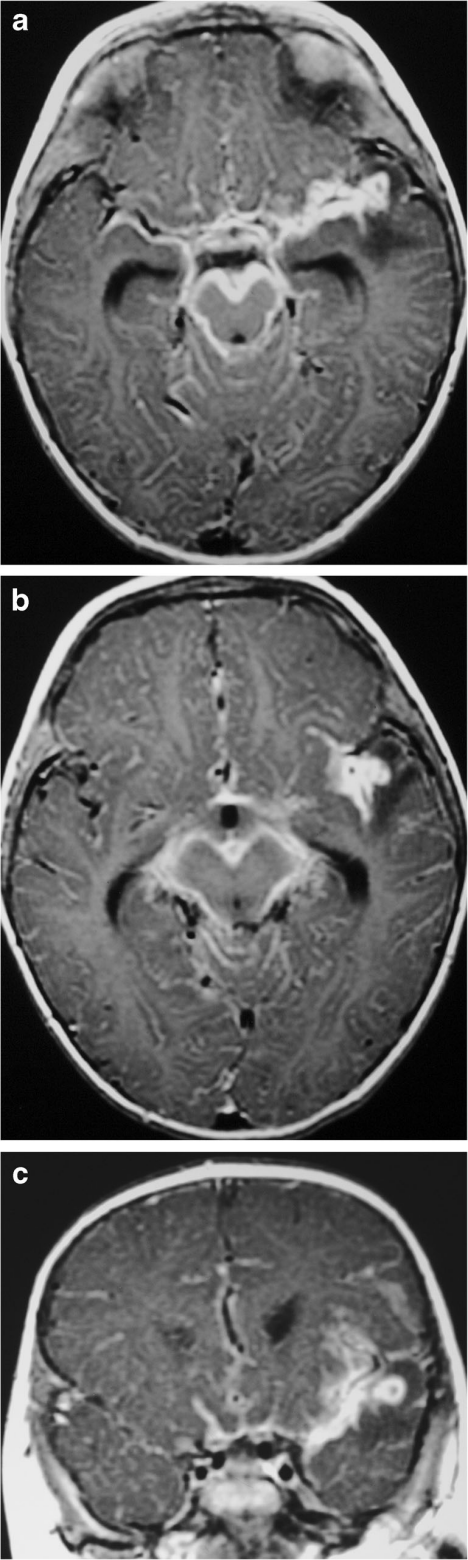
A 4-month-old baby boy with tuberculous meningitis. Axial (**a**, **b**) and coronal (**c**) post-contrast T1WI shows prominent extra-axial contrast-enhancement in the basal meninges, surrounding the brainstem, cerebellar sulci and Sylvian fissures. The leptomeningeal infection invades the left temporal lobe

*Mycobacterium tuberculosis* infection remains a major international concern, with increasing burden of disease in developed nations and young children being the most susceptible [56, 57]. MRI is increasingly being used to evaluate patients with suspected tuberculous meningitis, to aid in rapid early diagnosis [58]. MRI findings include basal leptomeningeal enhancement secondary to multiple granulomas and pial tuberculomas. The granulomas can be either non-caseating with homogeneous enhancement or caseating with rim enhancement (both T1 hypointense and T2 hyperintense) [59].

### Osteosarcoma (osteogenic sarcoma)

Rare malignant bone tumour comprised of proliferating neoplastic spindle-cells which produce osteoid and/or immature tumoural bone. They usually involve the endochondral bone-forming portions of the skull-base and are locally invasive with high metastatic potential. Over 75 % of osteosarcomas are diagnosed in patients between 8 and 25 years, and account for less than 1 % of all head and neck malignant tumours in the paediatric population [60]. They occur in children as primary tumours and adults associated with Paget’s disease, irradiated bone, chronic osteomyelitis, osteoblastoma, giant cell tumour and fibrous dysplasia. MRI will show a destructive lesion involving the skull base, with low-intermediate signal on T1WI, mixed low, intermediate or high signal on T2WI. Usually contains matrix mineralisation/ossification with low signal on T2WI and heterogeneous contrast enhancement.

### Ewing sarcoma

Bone tumour usually occurring between ages 5 and 30 years, with a greater prevalence in males and is the second most common primary bone malignancy affecting adolescents and young adults. It most commonly arises in the trunk and diaphysis of long bones, with only 4 % of cases within the head and neck region [61]. Tumours are locally invasive with a high metastatic potential. MRI will show a destructive lesion involving the skull base, with low-intermediate signal on T1WI, mixed low, intermediate or high signal on T2WI, with or without matrix mineralisation showing low signal on T2WI and heterogeneous contrast enhancement.

### Esthesioneuroblastoma (olfactory neuroblastoma) (Fig. 26)

**Fig. 26:**
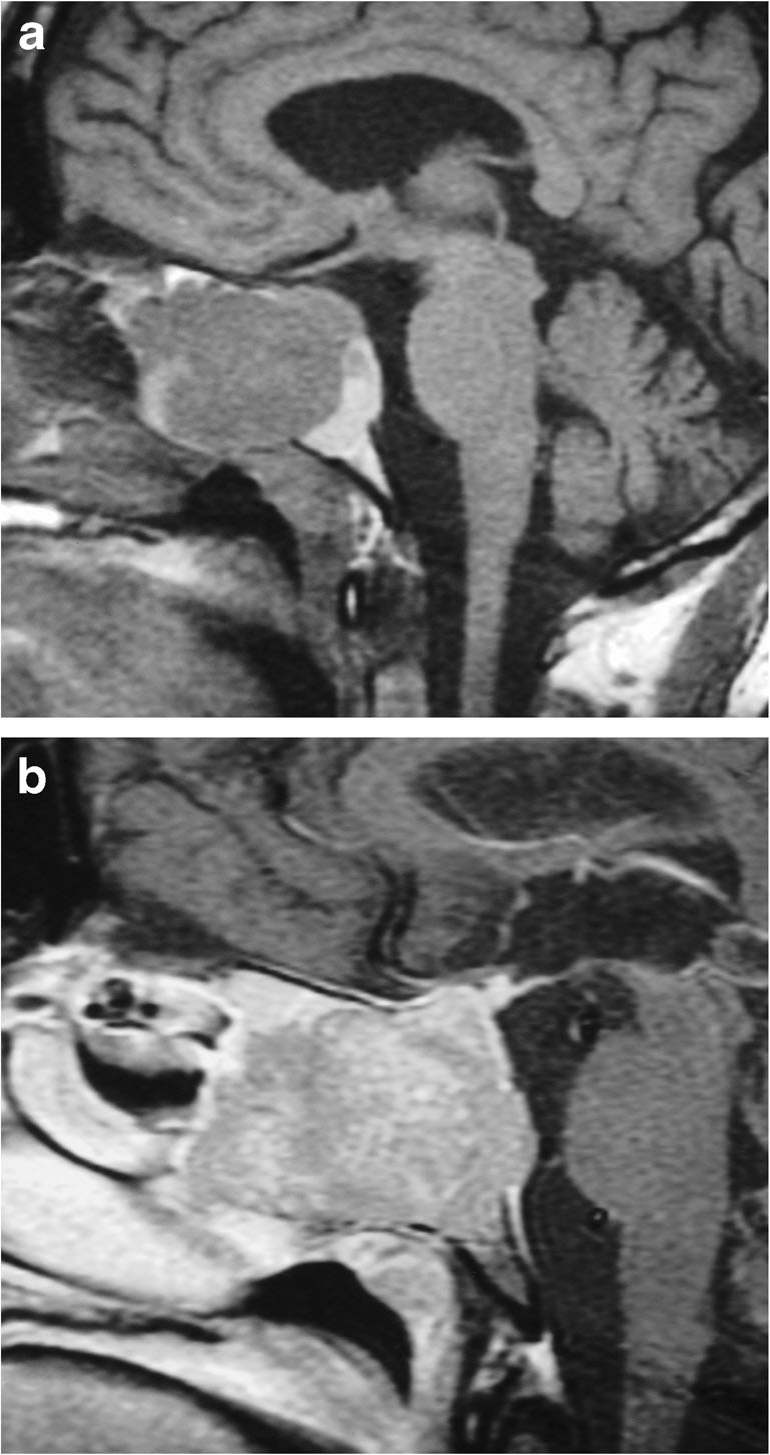
Enthesioneuroblastoma. Sagittal T1WI (**a**) and post-contrast T1WI (**b**) show a destructive mass lesion involving the sphenoid bone which invades the sphenoid sinus and sella. The tumour shows prominent, mildly heterogeneous contrast enhancement

These malignant neoplasms of neuroectodermal origin, arise from olfactory epithelium in the upper nasal cavity and cribiform region. Tumours consist of immature neuroblasts with variable nuclear pleomorphism, mitoses and necrosis. Tumour cells occur in aneurofibrillary intercellular matrix. There is a bimodal age of occurrence in adolescents (11-20 years) and adults (50-60 years) [62], with a greater prevalence in males. On MRI, they present as a locally destructive lesion with low-intermediate signal on T1WI, intermediate-high signal on T2WI and prominent contrast enhancement. They are commonly located within the superior nasal cavity, ethmoid air cells with occasional extension into other paranasal sinuses, orbits, anterior cranial fossa, and cavernous sinuses. Positron emission tomography (PET)/computed tomography (CT) can be useful for staging of disease and detection of metastases [63].

### Fibrous dysplasia (Fig. 27)

**Fig. 27:**
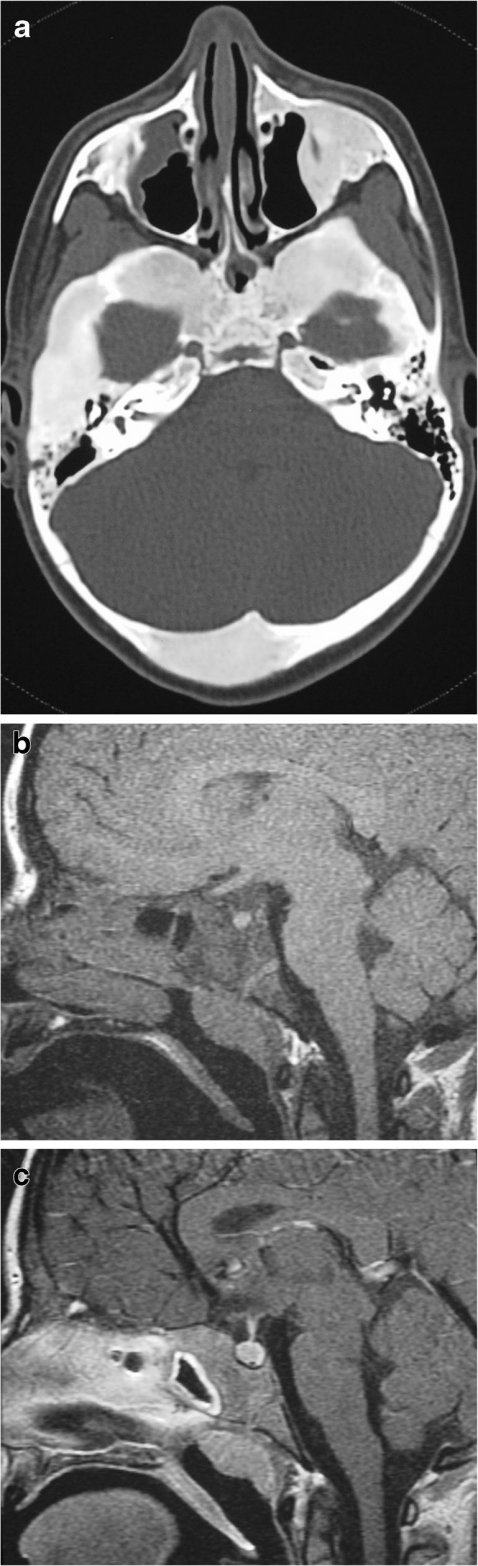
Fibrous dysplasia in a 5-year-old girl with McCune Albright syndrome. Axial CT (**a**) shows multiple expansile lesions involving the skull which have a “ground glass” appearance representing sites of polyostotic fibrous dysplasia. Sagittal T1WI shows expansion of the clivus which has diffuse low signal (**b**) with corresponding diffuse enhancement on sagittal post-contrast fat-suppressed T1WI (**c**)

Benign medullary fibro-osseous lesion, which can involve a single site (mono-ostotic) or multiple locations (poly-ostotic), results from developmental failure in the normal process of remodelling primitive bone to mature lamellar bone with zones of immature trabeculae within dysplastic fibrous tissue. Craniofacial bones are involved in 10-20 % of patients with the monostotic disease and in 50 % of patients with the polyostotic form [64]. Patients range in age from <1 year to the eighth or ninth decades of life with 75 % occurring before the age of 30 years. MRI features depend on the proportions of bony spicules, collagen, fibroblastic spindle cells, haemorrhagic and/or cystic changes, and if associated pathological fractures are present. Lesions are usually well-circumscribed and have low-intermediate signal on T1WI and proton-density weighted imaging (PDWI). On T2WI, lesions have variable mixtures of low, intermediate and/or high signal often surrounding by a low signal rim of variable thickness. Internal septations and cystic changes are seen in a minority of lesions. Bone expansion with thickened and/or thinned cortex can be seen. Lesions show contrast enhancement varying in degree and pattern [65].

### McCune-Albright syndrome

Refers to an uncommon disorder in which polyostotic fibrous dysplasia is associated with pigmented cutaneous macules (café-au-lait spots) with irregular indented borders, precocious puberty, and/or other endocrine disorders such as acromegaly, hyperthyroidism, hyperparathyroidism, and/or Cushing’s syndrome. McCune-Albright syndrome is uncommon and occurs in 2-3 % of patients with polyostotic fibrous dysplasia [66].

### Langerhans’ cell histiocytosis (Fig. 28)

**Fig. 28:**
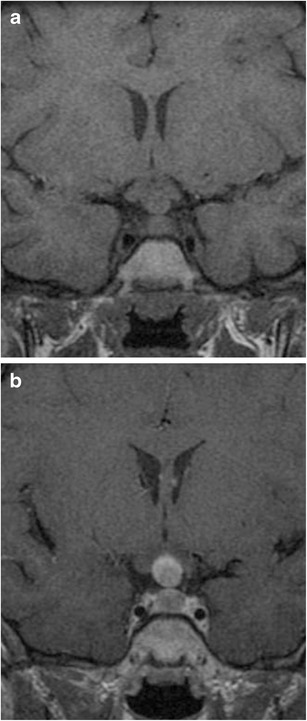
Langerhans cell histiocytosis in a 6-year-old girl with headaches and diabetes insipidus. Coronal T1WI shows a lesion involving the upper pituitary gland and stalk which has intermediate signal (**a**). The lesion shows prominent enhancement on post-contrast coronal T1WI (**b**)

Disorder of the reticuloendothelial system in which bone-marrow-derived dendritic Langerhans’ cells infiltrate various organs as focal lesions or in diffuse patterns. Langerhans cells have eccentrically located ovoid or convoluted nuclei within pale to eosinophilic cytoplasm. Lesions often consist of Langerhans cells, macrophages, plasma cells and eosinophils. Prevalence of 2 per 100,000 children less than 15 years [67], with only a third of the lesions occurring in adults. Localised lesions (eosinophilic granuloma) can be single or multiple in the skull, usually at the skull base. There is a striking preference for LCH to involve regions without a blood brain barrier, with the hypothalamic-pituitary axis the most frequently involved intracranial region [68], with patients often presenting with diabetes insipidus. MRI will typically show a fusiform or lobulated lesion with intermediate signal on T1WI and T2WI involving the pituitary stalk. The pituitary stalk is usually >3 mm in thickness [69], and is often associated with a loss of high signal on T1WI of the posterior pituitary. Lesions involving the pituitary usually show contrast enhancement [70].

### Lymphocytic hypophysitis (Fig. 29)

**Fig. 29:**
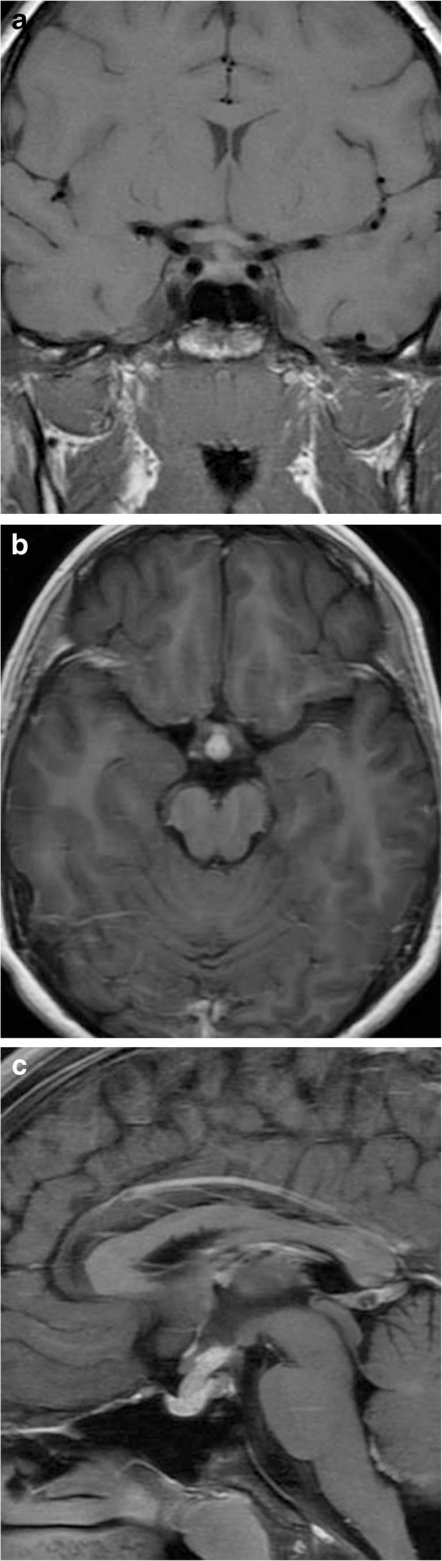
Lymphocytic hypophysitis in a 16-year-old boy who presented with frequent urination, which started when he was 9. Coronal T1WI (**a**) shows thickening of the pituitary stalk which has intermdiate signal. Post-contrast axial (**b**) and sagittal (**c**) T1WI shows thickening of the pituitary stalk with homogeneous enhancement

Rare autoimmune inflammatory process involving the pituitary gland confirmed by biopsy showing varying degrees of lymphocytic infiltration and plasma cells with fibrotic changes without multinucleated giant cells. They are more prevalent in women (80 %) who are in late pregnancy or postpartum but can also occur in children. Clinical findings include headaches and pituitary hormonal dysfunction, with deficiency of ACTH in adults and growth hormone in children [71]. Lesions can be associated with Hashimoto thyroiditis, polymyositis, pernicious anaemia, atrophic gastritis, psoriasis, systemic lupus erythematosus, adrenalitis and/or ovarian failure. MRI shows a slightly lobulated lesion with intermediate signal on T1WI, heterogeneous low-intermediate and high signal on T2WI involving the pituitary lobe with thickened pituitary stalk, and prominent homogeneous or heterogeneous contrast enhancement involving the pituitary gland, pituitary stalk and dura. A characteristic finding includes a parasellar T2 dark sign, which helps in determining lymphocytic hypophysitis from a pituitary adenoma [72].

### Rosai-Dorfman disease

A rare benign histiocytosis, also referred to as sinus histiocytosis with massive lymphadenopathy. It usually occurs in children and young adults, with a male-female ratio of 3:2 [73]. Patients may present with painless adenopathy with one or more extranodal sites. When there is intracranial involvement, patients may present with headaches, seizures, visual disturbances, numbness and/or paraplegia. Involvement of the central nervous system (CNS) occurs in 4 % and usually involves the intracranial or spinal dura, rarely occurring in the sella and suprasellar region. On MRI, suprasellar/sellar masses tend to be isointense on T1WI and hypointense/isointense on T2WI with homogeneous enhancement, and may be mistaken as a meningioma. A central hypointensiity on T2WI, thought to be related to free radicals release by inflammatory macrophages, may help distinguish from a meningioma [74, 75].

### Lymphomatous leptomeningial metastases (Fig. 30)

**Fig. 30:**
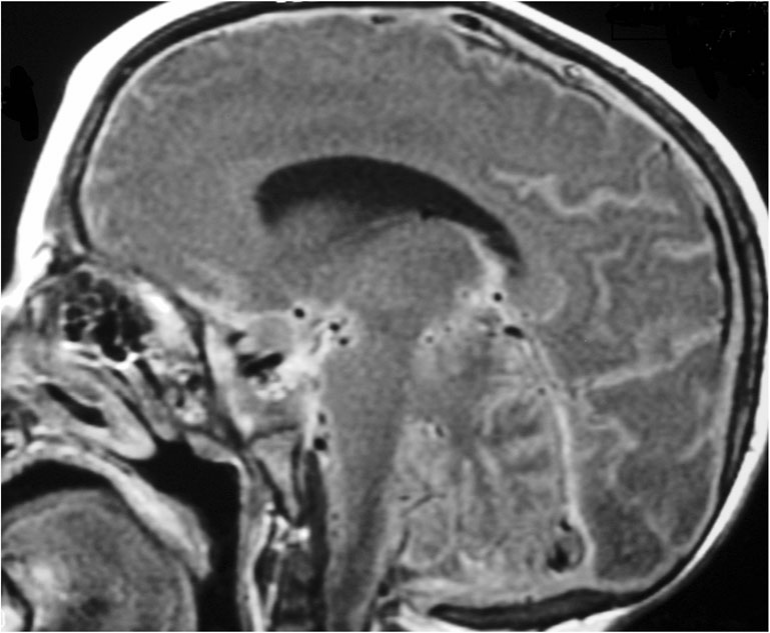
Leptomeningeal spread of lymphoma. Sagittal post contrast T1WI demonstrates diffuse intracranial and spinal leptomeningeal enhancement in a 22-month-old infant boy with lymphoma including the sella and parasellar subarachnoid space

Primary CNS lymphoma is extremely rare in children, and usually occurs as intra-axial lesions and infrequently as leptomeningeal tumour. Most cases are non-Hodgkin, high-grade B-cell lymphomas. There is a strong link in patients with AIDS or primary immunodeficiency [76]. Leptomeningeal metastases can also be seen with acute lymphoblastic leukaemia, medulloblastomas, germ-cell tumours, ependymomas and malignant gliomas [77].

### Vascular malformations (Fig. 31)

**Fig. 31:**
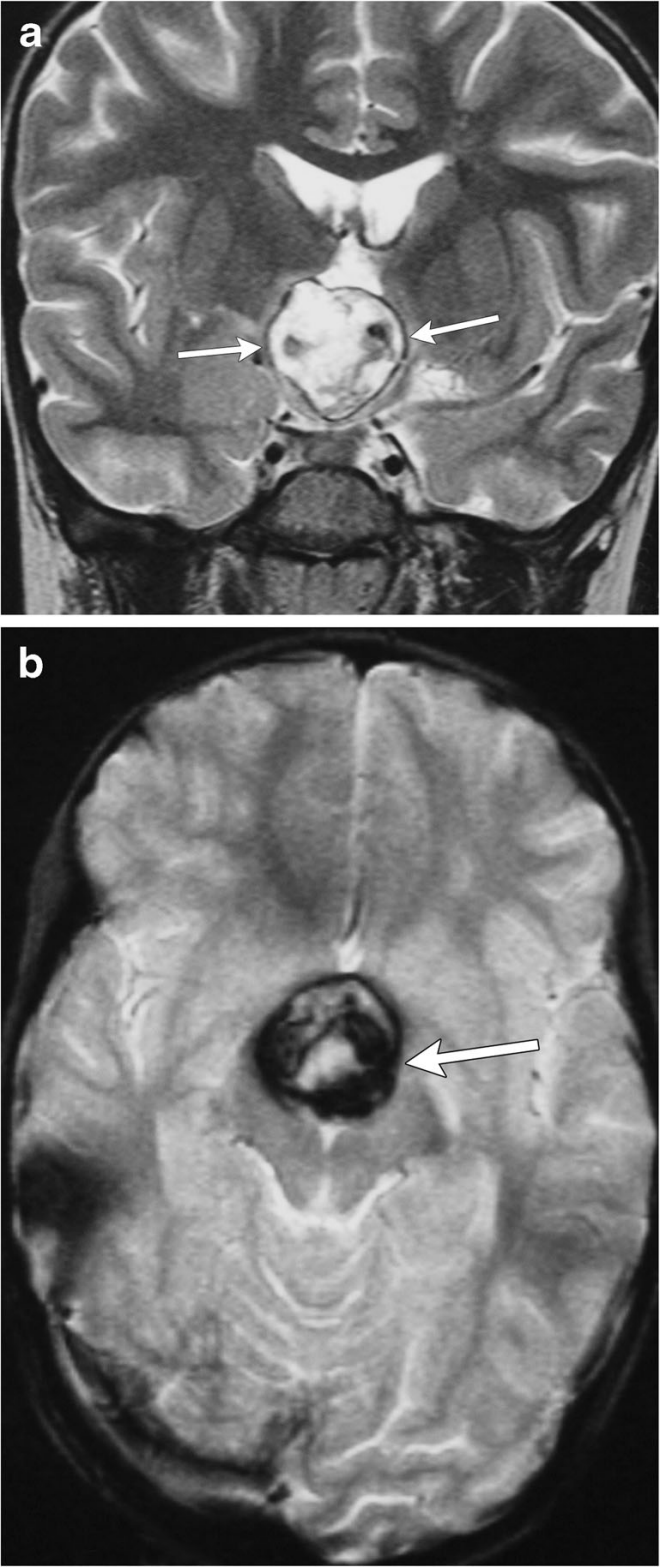
Cavernous malformation: coronal T2WI (**a**) and axial gradient echo (GRE) (**b**) images in a 5-year-old girl show a large suprasellar lesion with mixed low, intermediate and high signal intensity with a thin rim of low signal on T2WI and foci of low T2* signal from susceptibility effects of blood products on GRE

Numerous vascular malformations can be seen in the sellar/juxtasellar regions, including arterial aneurysms (saccular or giant aneurysms of the internal carotid artery [ICA]), cavernous carotid fistulas, arterial-venous malformations, haemangiomas or cavernous malformations. Cavernous malformations are the most common CNS vascular lesions in children, although the incidence is lower than in adults [78]. Compared to adults, these lesions are more aggressive in children with high rates of growth and haemorrhage, and a mean age of presentation of 9-10 years [79]. MRI findings will show single or multiple multilobulated intra-axial lesions with a peripheral or irregular zone of low signal on T2WI and T2* secondary to haemosiderin, with a central zone of variable signal on T1WI and T2WI depending on ages of haemorrhagic components. Contrast enhancement is usually absent.

## Conclusions

Imaging of the sellar and juxtasellar region in the paediatric population is ideally performed with MRI, and relies on a strong fundamental knowledge of the anatomy and signal characteristics of a normal gland and surrounding structures. The knowledge of key differentiating MRI characteristics in common and uncommon disease entities of the paediatric sellar and juxtasellar regions, in conjunction with the clinical findings, undoubtedly aids in alerting and guiding the clinician down the appropriate path of management. It is best to approach a suspected sellar or juxtasellar abnormality with an open mind and a broad differential, with MRI being the “gold standard” of imaging to accurately characterise these lesions.
